# Multilayered Human Activities Shape the Microbial Communities of Groundwater-Dependent Ecosystems on an Arid Oceanic Island

**DOI:** 10.1007/s00248-026-02797-0

**Published:** 2026-06-08

**Authors:** Francesco Di Nezio, Andrea Di Cesare, Marta García-Cobo, David Brankovits, Raffaella Sabatino, Giulia Borgomaneiro, Zacarías Fresno-López, Márcia Neunschwander Kurtz, Sarah Boulamail, Francesco Cozzoli, Lara Fumarola, Brett C. Gonzalez, Alvaro Roldán, Cristina Camacho, Alvaro García-Herrero, Leopoldo Moro, Concepcion Valdivia, Elena Mateo-Mederos, Guillermo García-Gómez, Diego Fontaneto, Gianluca Corno, Ester M. Eckert, Alejandro Martínez

**Affiliations:** 1 National Research Council of Italy (CNR) – Water Research Institute (IRSA), Verbania, Italy; 2 National Biodiversity Future Center (NBFC), Palermo, Italy; 3 Department of Biodiversity, Ecology and Evolution, Universidad Complutense de Madrid, Madrid, Spain; 4 National Research Council of Italy (CNR) – Research Institute on Terrestrial Ecosystems (IRET), Lecce, Italy; 5 Department of Biological and Environmental Sciences and Technologies (Di.S.Te.B.A), University of Salento, Lecce, Italy; 6 School of Biological Sciences, The University of Western Australia, Minderoo-UWA Deep-Sea Research Centre, Perth, Australia; 7 Technical and Scientific diving freelance, Las Breñas, Yaiza, Spain; 8 Freelance, Puerto del Carmen, Tías, Las Palmas, Spain; 9 Asesores Técnicos de Medio Ambiente ATECMA SL, Collado Villalba, Spain; 10 Servicio de Biodiversidad, Gobierno de Canarias, Santa Cruz de Tenerife, Spain; 11 Lanzarote and Chinijo Islands UNESCO Global Geopark, Mozaga, Spain; 12 Rey Juan Carlos University, Global Change Research Institute (IICG), Madrid, Spain

**Keywords:** Microbiome, Groundwater, Pathogens, Anthropogenic influence, Aquifer, Biodiversity

## Abstract

Island coastal aquifers, though spatially limited, sustain key ecosystem functions linked to locally critical provisioning, maintenance and cultural ecosystem services. These functions are largely dependent on the presence of highly adapted biological communities, whose microbial components remain poorly understood. Here, we describe bacterial communities across groundwater-dependent ecosystems on Lanzarote (Canary Islands, Spain), spanning habitats with contrasting environmental conditions and degrees of human influence, using 16 S rRNA gene amplicon sequencing. We then infer the processes shaping community variation by integrating diversity partitioning, indicator species analysis, and machine-learning classification. Bacterial taxonomic diversity varied significantly among habitats, with community composition primarily structured by turnover, consistent with environmental filtering. In contrast, predicted human-associated and potentially pathogenic taxa showed patterns dominated by nestedness, indicating localized enrichment linked to anthropogenic inputs. Caves, enclosed marine bays, and hypersaline systems hosted the most compositionally distinct microbial communities, whereas wells and anchialine pools showed greater overlap in community composition. Together, our results suggest that groundwater microbial communities are influenced by the interplay between environmental filtering and anthropogenic inputs, and that coastal aquifers can act simultaneously as reservoirs of natural biodiversity and sinks of human-associated bacteria. These findings highlight the need for integrative monitoring and conservation strategies that incorporate both hydrological and biological components to safeguard groundwater-dependent ecosystems on oceanic islands.

**Supplementary Information:**

The online version contains supplementary material available at 10.1007/s00248-026-02797-0.

## Introduction

Throughout time, islands have been both cradles and reservoirs of biodiversity [1]. Although they account for only 5.3% of Earth’s land surface, islands not only harbor roughly 20% of all described species, with exceptionally high endemism rates, but also 27% of human languages [2, 3]. The persistence of both ecosystems and human societies on islands critically depends on groundwater stored in coastal aquifers, which often represent the primary source of freshwater for drinking and irrigation. Beyond provisioning services, these aquifers sustain vegetation and wetlands, buffer drought effects, and support coastal ecosystems through submarine groundwater discharge, which delivers nutrients and trace elements to the ocean and contributes to coastal food webs [4–8].

Island aquifers are typically stratified, with a meteoric freshwater lens overlying denser seawater. At their interface, a subterranean estuary develops, creating a biogeochemical reaction zone that modulates groundwater chemistry before its discharge into the coastal sea [9, 10]. These systems generate strong gradients in salinity, oxygen, and nutrients, which extend across interconnected groundwater-dependent habitats such as caves, anchialine pools, springs, and coastal discharge zones [11–14]. These habitats, collectively referred to as anchialine systems, are characterized by steep physicochemical gradients and varying degrees of hydrological connectivity, and host highly specialized and often endemic fauna, including crustaceans and other stygobitic organisms adapted to resource limitation, darkness, and environmental stability.

Microbes play a key role in sustaining nutrient and carbon cycling, transferring carbon and energy to the higher trophic levels of the anchialine food web [15–17]. In hypersaline areas, halophilic archaea and halotolerant bacteria employ compatible-solute accumulation or “salt-in” ion-balancing strategies to withstand osmotic stress [18, 19]. In springs, anchialine pools and sinkholes, phototrophs, like rhodopsin-bearing prokaryotes, harvest light. In the darkness of caves, bacterial communities rely on limited allochthonous organic carbon inputs or on in situ carbon fixation by chemolithotrophic microbes utilizing reduced sulfur, methane, nitrogen, or other inorganic compounds [20–22].

Human activities profoundly reshape groundwater microbial communities by altering both environmental conditions and sources of microbial input. Coastal aquifer systems are particularly vulnerable to such pressures: at local and regional scales, tourism, agriculture, and urbanization intensify groundwater extraction, nutrient enrichment, and contamination [23–25], while at broader scales, climate change and sea-level rise promote warming, saltwater intrusion, and hydrological stress [26, 27]. These drivers favor fast-growing, human-associated taxa, including opportunistic pathogens, while reducing environmentally specialized lineages. At the same time, hydrological connectivity can facilitate the transport of surface-derived microorganisms into subsurface systems, leading to local enrichment or homogenization of microbial assemblages. Together, these processes generate distinct ecological signatures, including increased nestedness driven by the addition of human-related taxa, or turnover driven by environmental filtering across contrasting habitats.

Because microbial communities respond rapidly to environmental change, they are sensitive indicators of shifts in water quality and geochemical stability. Rising ocean and groundwater temperatures can enhance microbial activity [28], while reduced oxygen solubility exacerbates hypoxia and anoxia, favoring anaerobic and potentially pathogenic taxa [29, 30]. Moreover, a substantial fraction of known pathogens may gain ecological advantages under future climate scenarios [31, 32]. In combination with nutrient enrichment and hydrological disturbance, these processes are likely to accelerate ecosystem degradation and increase health risks through groundwater contamination by pathogens and fecal bacteria [33, 34]. These scenarios highlight the urgent need for baseline microbial datasets, early warning systems, and informed policy interventions [35]. Yet, many aquifers still lack a robust characterization of their microbial communities, limiting our ability to design effective research and conservation strategies.

The goal of this paper is twofold: first, we describe bacterial communities across groundwater–dependent ecosystems on Lanzarote (Canary Islands, Spain), spanning a gradient of anthropogenic influence; second, we test how different habitats—each associated with distinct environmental conditions and human uses—shape community composition and structure, with particular emphasis on potentially pathogenic, human-derived, and environmentally derived taxa. We selected Lanzarote because the low-altitude coastlines expose the basal aquifer through lava tubes and anchialine pools, whereas perched aquifers are easily accessible through springs and wells. These waters have long underpinned human settlement, navigation and coastal trade, and support a uniquely diverse anchialine fauna; however, accelerating population growth, together with pollution, rising water demand, and urban expansion, now threatens Lanzarote’s groundwater ecosystems [36]. We hypothesize that bacterial communities are structured by two non-mutually exclusive processes: environmental filtering driven by habitat-specific physicochemical conditions, and anthropogenic inputs introducing human-associated taxa into the aquifer system. From this framework, we derived three specific predictions. First, the composition of the overall bacterial community will differ among habitats and be primarily structured by turnover, reflecting environmental selection across contrasting conditions. Second, potentially pathogenic and human-associated taxa will show habitat-specific enrichment linked to anthropogenic influence and hydrological connectivity, with compositional differences dominated by nestedness. In contrast, environment-associated taxa will mirror the overall community and remain turnover-dominated. Third, habitats that differ strongly in environmental conditions or anthropogenic influence will host more compositionally distinct bacterial communities, resulting in uneven discriminability and distinct sets of indicator taxa. To address these hypotheses, we adopt a stepwise analytical framework that progresses from descriptive characterization of community composition to formal statistical inference of habitat-associated differences, and finally to the identification of taxa driving these patterns. By involving both researchers and stakeholders in our team, we translate these results into a set of consensus-based strategies aimed at enhancing the monitoring and conservation of Lanzarote’s groundwater-dependent habitats.

## Materials and Methods

### Study Sites and Sampling Design

**Fig. 1:**
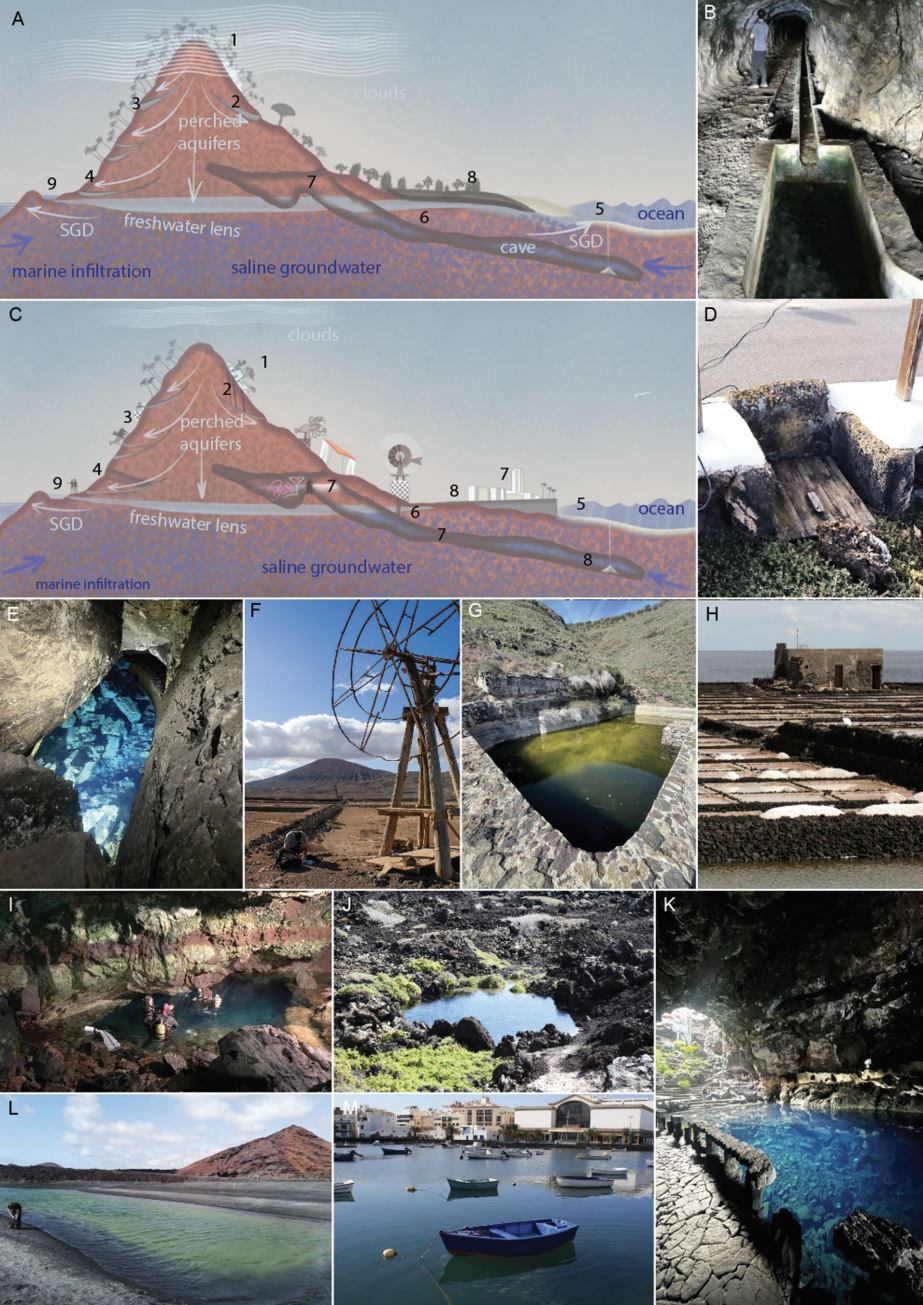
Human-driven impacts on the island aquifer and groundwater and groundwater-dependent ecosystems sampled during this study. **(a)** Pristine conditions, showing the recharge of the aquifer driven by Tradewind precipitation and hypothetical water flow from highlands to the island’s basal aquifer—partially interrupted by aquitards forming perched aquifers. **(b)** Famara water mine, accessing the lower part of the brackish perched aquifer of Famara **(c)** Current state. Urbanization (1) reduces natural thermophilic forest cover and aquifer recharge; (2) wells and cesspits associated with urban areas extract and potentially contaminate perched aquifers; (3) springs associated to aquitards are modified for agriculture and livestock, others (4) are exploited for water mining; (5) coastal development disrupts submarine groundwater discharge, and potentially introduces anthropogenic inputs via illegal wastewater discharge; (6) basal aquifer is exploited via wells for salt production; (7) anchialine caves are altered for tourism; (8) coastal infrastructure degrades vegetation and dune systems, affecting the carbon input into the aquifer; (9) increased population and tourism degrade anchialine pool water quality. **(d)** Urban well in Haría, accessing the brackish perched aquifer of Famara. **(e)** Cueva de los Lagos, a saline, dark, anchialine cave, part of La Corona lava tube. **(f)** Well associated with the saltworks of Los Agujeros, pumping saline groundwater from the island’s basal aquifer into the saltpans for salt production. **(g)** Fuente del Chafariz, a freshwater spring-fed pool, influenced by rainfall and terrestrial runoff and frequently accessed by livestock. **(h)** Saltpans of Salinas de los Agujeros (wikicommons) **(i)** Túnel de la Atlántida, a saline, artificially illuminated section of La Corona lava tube, part of Los Jameos del Agua touristic center. **(j)** Charcos de Luis, a saline, illuminated, anchialine pool in the north of the island, occasionally visited by tourists. **(k)** Los Jameos del Agua, a saline, illuminated anchialine cave, part of Los Jameos del Agua touristic center, within La Corona lava tube. **(l)** Charco Grande de Montaña Bermeja, a saline, illuminated, anchialine pool in the south of the island, often visited by tourists. **(m)** Charco de San Ginés, a modified coastal bay where docks infrastructure has altered submarine groundwater discharge, frequented by fishermen and potentially impacted by pollution by surrounding residential areas. (SGD = Submarine Groundwater Discharge)

To test the hypothesis that different groundwater-dependent habitats harbor distinct bacterial communities, we sampled the main accessible aquifer-related habitats of Lanzarote, including lava-tube caves, anchialine pools, saltworks, and groundwater wells and galleries. Because these habitats are unevenly distributed across the island and differ in accessibility [37], sampling effort across habitat types was necessarily unbalanced. Table S1 summarizes the aquifer compartments, representative habitats, ecosystem functions, and associated anthropogenic impacts considered in this study (Fig. 1).

We sampled 19 sites representing six habitat types on Lanzarote (Supplementary Text 1, Figure S1, Table 1). In total, we collected 28 samples, as Túnel de la Atlántida, Jameos del Agua, Cueva de los Lagos, and Caletón Blanco were sampled at multiple sections. We collected water using sterile 5 L containers or sterile Ziplock bags during cave dives in the lava tube. Before use, we sterilized all collection materials with 5% chlorine and rinsed with sterile distilled water prior to use. We processed all samples within 24 h of collection.

**Table 1:** Sampling sites across Lanzarote, including site names, sample IDs, habitat classification, geographic coordinates (WGS84), salinity, light conditions, DNA concentration, and SRA accession numbers. Salinity was measured using a YSI multiparameter probe; values marked with a tilde (~) were estimated from seawater, whereas those indicated with a single asterisk (*) were measured using a refractometer. Values marked with double asterisks (**) were retrieved from Wilkens et al. [39]. As all coastal sites are influenced by tidal dynamics, salinity values may vary temporally (see [39])

Site	Sample ID	Habitat	Latitude	Longitude	Salinity	Light	DNA concentration (ng µL^− 1^)	SRA accession number
Cueva de los Lagos - first lake	CUL1	Cave	29.1577	−13.4366	35.7‰	Dark	11.41	SRR36511595
Cueva de los Lagos - sump entrance	CUL2	Cave	29.1575	−13.4343	35.7‰	Dark	0.19	SRR36511572
Los Jameos del Agua - Jameo Grande	JDA_pool 1 (*upstream*)	Cave	29.1573	−13.4309	35.6‰	Twilight	0.20	SRR36511584
Los Jameos del Agua - middle	JDA_pool 2 (*center*)	Cave	29.1573	−13.4309	35.6‰	Twilight	1.71	SRR36511568
Los Jameos del Agua - Jameo Chico	JDA_pool 3 (*downstream*)	Cave	29.1573	−13.4309	35.6‰	Twilight	1.61	SRR36511596
Túnel de la Atlántida - entrance	TDA_entrada	Cave	29.1571	−13.4303	36.5‰	Twilight	0.14	SRR36511590
Túnel de la Atlántida - La Sima	TDA_sima	Cave	29.1564	−13.4281	36.5‰	Dark	0.16	SRR36511573
Túnel de la Atlántida - Lago Escondido	TDA_LE	Cave	29.1565	−13.4291	36.5‰	Dark	0.45	SRR36511567
Túnel de la Atlántida - Montaña de Arena	TDA_MA	Cave	29.1575	−13.4343	36.5‰	Dark	4.77	SRR36511579
Charcos de Luis (Orzola)	CHL	Anchialine pool	29.2022	−13.4231	40.0‰**	Light	38.49	SRR36511594
Charco de Punta Prieta (Punta Prieta)	CHP	Anchialine pool	29.2011	−13.4211	36.5‰*	Light	28.62	SRR36511592
Charco de Suso (Punta Mujeres)	CHS	Anchialine pool	29.1518	−13.4389	38.0‰	Light	3.20	SRR36511588
Charco de Zac (Caleta de las Escamas)	CHZ	Anchialine pool	29.1728	−13.4266	36.5‰*	Light	12.10	SRR36511593
Charco Grande de Montaña Bermeja (El Golfo)	MBB	Anchialine pool	28.9612	−13.8297	38.2‰	Light	2.91	SRR36511577
Charco Pequeño de Montaña Bermeja (El Golfo)	MBS	Anchialine pool	28.9617	−13.8266	36.1‰	Light	2.23	SRR36511578
Salinas de los Agujeros - saltpans (Guatiza)	CSS 1, 2	Saltworks	29.0604	−13.4614	95.8‰	Light	24.22 47.81	SRR36511583
Salinas de los Agujeros - well (Guatiza)	CSP	Saltworks	29.0608	−13.4638	36.0‰*	Twilight	46.12	SRR36511570
Caletón Blanco - outer bay (Orzola)	CBL	Enclosed marine bay	29.2175	−13.4424	~ 36‰	Light	0.52	SRR36511586
Caletón Blanco - inner bay (Orzola)	CBC 1, 2	Enclosed marine bay	29.2187	−13.4444	~ 36‰	Light	1.43 0.95	SRR36511585 SRR36511587
Charco de San Ginés (Arrecife)	CHG	Enclosed marine bay	28.9612	−13.5482	36.7‰	Light	2.47	SRR36511580
Punta del Usaje (La Corona)	JDA_beach 1 (*littoral*)	Enclosed marine bay	29.1565	−13.4267	~ 36‰	Light	51.01	SRR36511589
Punta del Usaje (La Corona)	JDA_beach 2 (*inland*)	Enclosed marine bay	29.1565	−13.4267	~ 36‰	Light	22.20	SRR36511591
Casa del Agua de Famara	FWH	Well	29.1402	−13.5255	2.6‰	Dark	2.79	SRR36511575
Galería de Famara	FWM	Well	29.1442	−13.5248	2.6‰	Dark	6.43	SRR36511576
Galería del Pescador (Famara)	GAF	Well	29.1403	−13.5243	3.6‰	Twilight	3.55	SRR36511574
Fuente del Chafaris (Tabayesco)	FUC	Spring	29.1231	−13.5084	0.9‰	Light	42.29	SRR36511581
Pozo de Suso (Haría)	SHW	Well	29.1446	−13.5029	1.0‰	Dark	0.68	SRR36511569
Galería de la Fuente del Chafaris (Tabayesco)	TAB	Well	29.1231	−13.5084	0.9‰	Twilight	0.69	SRR36511571

We characterized environmental context using salinity at each sampling site during fieldwork (Table 1). We did not systematically measure additional hydrochemical parameters (e.g., nutrients, dissolved oxygen, or trace elements) during this sampling campaign. Accordingly, we focus on patterns of microbial community composition across habitat types rather than on detailed physicochemical drivers of community assembly.

### Laboratory Procedures and Sequencing

We filtered approximately 1–5 L of water per sample through sterile 0.22 μm pore-size polycarbonate filters (Merck Millipore, Germany) using a peristaltic pump until clogging. We then stored filters at − 20 °C until DNA extraction. Prior to extraction, we cut each filter into three equal sections and processed each section independently to maximize DNA recovery. We then pooled the DNA extracts, resulting in a single DNA sample per environmental sample (*n* = 28).

We extracted DNA using the DNeasy UltraClean Microbial Kit (QIAGEN, Hilden, Germany), following the manufacturer’s instructions. We measured DNA using a Qubit 1X dsDNA High Sensitivity Assay kit (Thermo Fisher Scientific, Waltham, USA). DNA concentrations ranged between approximately 0.1 and 51.0 ng µL⁻¹ (Table 1) across samples. We subsequently stored all DNA samples at − 20 °C until further processing. We regularly perform sample-free DNA extractions and sequence these negative controls in the laboratory (approximately every 48th extraction). Those negative controls did not produce detectable amplicons under standard PCR conditions.

For 16S rRNA gene amplicon sequencing, we shipped samples under controlled conditions to an external service provider (IGA Technology Services, Udine, Italy). Amplicon libraries targeting the V3–V4 hypervariable region of the 16S rRNA gene were prepared following the Illumina 16S Metagenomic Sequencing Library Preparation protocol in two amplification steps. The first PCR targeted the V3–V4 region using primers 341 F (5’-CCTACGGGNBGCASCAG-3’) and 805R (5’-GACTACNVGGGTATCTAATCC-3’) [38] and consisted of 25 amplification cycles. A second index PCR was subsequently performed to attach dual indices and Illumina sequencing adapters and consisted of 8 amplification cycles. Libraries were sequenced on a Aviti platform (Element Biosciences, San Diego, CA) using 300-bp paired-end reads. Additional details on PCR conditions, library preparation, and sequencing are provided in Supplementary Text 2.

### Sequence Processing and Dataset Assembly

Base calling, demultiplexing, and adapter masking were performed using Bases2fastq software v.1.8 (Element Biosciences). Raw sequences were processed using the DADA2 pipeline (v. 1.36.0) following the online tutorial [40] in R v. 4.5.0 [41]. Forward and reverse reads were truncated at 280 bp and 220 bp, respectively, after primer trimming. Quality-filtering parameters are provided in Supplementary Text 2. Taxonomic assignment was performed against the SILVA database (v138.2).

The dataset was refined by removing ASVs identified as archaea (359 ASVs, 3.75% of the reads), mitochondria (21 ASVs, 0.02% of the reads), chloroplasts (176 ASVs, 2.46% of the reads), or unassigned at the phylum level (369 ASVs, 0.68% of the reads). Archaeal sequences were excluded because the primers used (341 F/805R) are optimized for bacterial 16S rRNA amplification and provide only partial coverage of archaeal diversity. ASVs unassigned at the phylum level were considered likely artifacts or outside the scope of this study (Table S2). Subsequently, we excluded rare ASVs defined as those occurring in fewer than two samples or represented by fewer than three reads. The same filtering procedure was applied consistently across all samples, allowing for reliable comparisons among habitats. The resulting dataset, containing all remaining ASVs regardless of the taxonomic rank at which they were identified during the pipeline (e.g., family or genus), hereafter referred to as the ASV table.

### Taxonomic Composition and Richness Across Lanzarote Groundwater-Related Ecosystems

To describe the taxonomic composition of bacterial communities across samples, we aggregated ASVs according to their taxonomic assignments by summing read counts across ASVs sharing the same taxonomic label at the focal rank by SILVA (e.g., genus). Only taxa identified to at least the focal rank and represented by ≥ 50 reads in the dataset were retained for composition analyses. Consequently, taxa reported as “unclassified” in downstream analyses refer to sequences that were successfully assigned to higher taxonomic ranks (e.g., phylum, class, or order) but could not be confidently classified at the focal rank (e.g., ASVs not identified to family, genus, or species ranks when family, genus, or species composition of the samples is addressed).

To assess how potentially pathogenic, human-derived, and environmental bacterial communities vary across habitats, ASVs identified at least to genus level were annotated using two pipelines:

1. We annotated pathogenic potential based on the nomenclature and taxa-related information on pathogenicity levels from the *List of Prokaryotic names with Standing in Nomenclature* (LPSN; https://lpsn.dsmz.de/*)* accessed between the 2nd and the 10th of September 2025 (Table S3). We defined the pathobiome as the total set of taxa with potential or confirmed pathogenicity in humans or animals (Risk Group 2 and 3 in the LPSN) [42, 43]. This approach relies on amplicon sequencing data and therefore limits inference to potential pathogenicity rather than confirmed pathogenic activity. Future studies should integrate cultivation-dependent and functional approaches to characterize pathogenic strains, while acknowledging that these methods capture only the cultivable fraction of bacterial diversity.
2. We classified ecological origin based on predominant habitat information compiled from over 4,000 reference entries (metadata related to bacterial isolates, MAGs, and metagenomes recovered from articles published in Q1 journals in the fields of Ecology and Microbiology from 2020) by literature mining using *scite_*AI (https://scite.ai/home) and Copilot AI based on the GPT-5 model by OpenAI, available to employees of the National Research Council of Italy (CNR) (https://copilot.microsoft.com/*)* and manual validation (see Supplementary Text 3 for the detailed query framework and decision workflow). Outputs from the two meta-searches were considered valid only when perfectly matching. In the case of discrepancies, the entries were manually reviewed and assigned based on available bibliographic evidence; when such evidence was insufficient, the entry was categorized as ‘unassigned’. Furthermore, 10% of the matching output was randomly selected for manual cross-validation, resulting in a 100% agreement rate. This agreement reflects consistency of the classification workflow rather than independent validation accuracy. Taxa for which ≥ 90% of reference hits were recovered from natural environments (oceans, lakes, rivers, sediments, soils, plants, animals) were defined as of environmental origin (E), whereas taxa for which ≥ 90% of hits derived from humans or human-constructed environments (human microbiome, hospitals, wastewaters, sludges) were considered of anthropogenic origin (W). Taxa where this threshold was not reached by any of the two categories, or taxa where it was not possible to define any origin (≥ 90%) were classified as ubiquitous/unclassified (O) (Table S3). This classification framework allowed an ecological interpretation of the pathobiome (potential pathogens belonging to Risk Group 2–3) in relation to anthropogenic influence. This classification remains approximate, as it is on the one hand limited by the quality and rank of our taxonomic identification and, on the other hand, biased by the uneven information available on the ecological preference of the genera identified in our study.

### Statistical Inference

Our overarching hypothesis was that habitats host distinct bacterial assemblages that differ in richness and composition due to two non-mutually exclusive processes: (i) environmental selection associated with contrasting aquifer conditions, expected to favor turnover among habitats and (ii) anthropogenic disturbance, expected to introduce or selectively favor/remove human-associated lineages and thereby increase nestedness.

To disentangle these processes, we tested three sub-hypotheses. H1: Taxonomic richness and composition of the whole bacterial community differ among habitats, with compositional differences dominated by turnover. H2: Potentially pathogenic and human-associated fractions show habitat-specific enrichment and β-diversity dominated by nestedness, whereas the environmental fraction is expected to mirror the whole community and remain turnover-dominated. H3: Bacterial communities exhibit habitat-dependent compositional differences, reflecting variation in the strength of environmental filtering and anthropogenic influence across the system.

Sampling was unbalanced among habitats, reflecting the naturally uneven availability and accessibility of aquifer-related habitats on Lanzarote, with some habitat types represented by fewer samples than others [37]. We retained this structure in the analyses and assessed adequacy and fit for each statistical model, through diagnostic checks of residual distribution, dispersion, homoscedasticity, collinearity, and influential observations. We interpreted habitat effects based on the fitted models and post hoc pairwise contrasts, while acknowledging reduced power and increased uncertainty for poorly represented habitats.

In addition to the main analyses, we conducted supplementary analyses to further characterize (i) phylogenetic diversity patterns across habitats and (ii) fine-scale variation within La Corona lava tube system (Cueva de los Lagos, Jameos del Agua, and Túnel de la Atlántida). These analyses included additional diversity metrics, model outputs, and subsite-level comparisons, and are presented in detail in Supplementary Text S4 and Tables S8–S9.

#### Patterns of Taxonomic Diversity of the Bacterial Community Across Habitats

To test our first hypothesis — that bacterial community composition differs among habitats, and that between-habitat dissimilarity is dominated by turnover — we analyzed the α- and β-taxonomic diversity of the ASV table, without taxonomic aggregation, to avoid biases associated with incomplete taxonomic coverage in the reference database (i.e. SILVA v. 138.2).

α-taxonomic diversity was quantified as taxonomic richness (number of unique ASVs per sample) and Shannon diversity, calculated using the function ‘diversity’ in the R package *vegan* (v. 2.6–8) [44]. β-taxonomic diversity was quantified as differences in ASV composition across samples using the Bray–Curtis dissimilarity index on the community matrix with abundances normalized by total reads per sample. β-diversity was calculated and partitioned into its nestedness and turnover components using the function ‘beta’ in the R package *BAT* (v. 2.9.5) [45].

Predictors included habitat type and continuous covariates representing (i) environmental context: ‘distance from the sea’ (m), ‘light’ (categorical: dark, twilight, light), ‘salinity’ (‰), ‘altitude’ (m) and (ii) anthropogenic context: ‘number of buildings within a 1-km radius’, ‘total road length within a 1-km radius’ (m). The number of reads was included in the alpha taxonomic models to account for uneven sequencing depth.

We assessed collinearity among predictors using the pairs.panels function from the R package *psych* (v. 2.4.1), using a threshold of |Pearson r| > 0.7. Based on this screening, we retained distance from the sea as a proxy for environmental variation and the number of buildings within a 1-km radius as a proxy for anthropogenic pressure, while controlling for sequencing depth.

To test the relationship between taxonomic richness and environmental predictors, we modelled ASV richness with generalized linear models with a negative-binomial error distribution and log link (function ‘glm.nb’; R package *MASS*, v. 7.3 [46]), to account for overdispersed count data, and Shannon diversity using linear models (function lm; R package *stats*). Model assumptions and fit were evaluated with the R package *performance* v. 0.7.3 [47], including checks for residuals distribution, homoscedasticity, multicollinearity, and influential observations [48]. Since both models included both categorical and continuous predictors, we summarized model effects using type II ANOVA tables (function Anova; R package *car* (v. 3.0.10) [49]), which assess the significance of each predictor using Wald χ² tests. Pairwise differences among habitats were evaluated using estimated marginal means with Tukey-adjusted comparisons (function emmeans, R package *emmeans* v. 1.10.7) [50].

We tested the association between predictors and community dissimilarity on overall Bray–Curtis dissimilarity matrix using PERMANOVA (function adonis2; R package *vegan* v. 2.6–8), using the same set of covariates as in the richness model. Homogeneity of multivariate dispersions among habitat types was evaluated using the betadisper function in the *vegan* R package to ensure that significant results were not driven by differences in dispersion.

Differences in richness across habitats were visualized using dot plots (*n* < 4) and boxplots (*n* ≥ 4) in *ggplot2* [51], depending on sample size. Similarity among samples was explored using non-metric multidimensional scaling (nMDS; function metaMDS in *vegan*) and hierarchical clustering with average linkage (function ‘hclust’, method = “average”; R *base* v. 4.3.2) [52]. The distribution of β-diversity values within and among habitat types was visualized using density plots with median values, providing a more comprehensive representation of dispersion and group overlap than summary statistics alone.

#### Patterns of Taxonomic Diversity of Potentially Pathogenic, Human-Associated and Environmental Bacteria Across Habitats

To test our second hypothesis—that potentially pathogenic and human-associated bacteria show habitat-specific enrichment and β-diversity dominated by nestedness, whereas the potentially environmental taxa are primarily structured by turnover—we analyzed patterns of taxonomic diversity across these subsets following the same structure described above (2.5.1).

Because our focus was on enrichment relative to the total community rather than absolute richness or abundance, we modelled proportional responses. Specifically, proportional abundance (i.e. the number of reads assigned to each functional group relative to the total reads per sample) was analyzed using beta-binomial generalized linear models implemented using the function ‘glmmTMB’ in the R package *glmmTMB* [53]. This formulation accounts for the overdispersion commonly observed in count-based proportional data. Proportional richness (i.e. the number of taxa within each functional group relative to total richness per sample) was modelled using binomial generalized linear models implemented with the function ‘glm’ in base R. Both proportional abundance and richness were expressed as two-column matrices of successes and failures (e.g. focal group vs. rest of the community), allowing direct modelling of proportions.

We applied the same predictors and diagnostics procedures as described in 2.5.1. Model parameters were evaluated using Wald χ² tests via type II ANOVA (Anova, *car* v. 3.0.10). Pairwise differences among habitat types were assessed using estimated marginal means with Tukey-adjusted comparisons (emmeans, emmeans v. 1.10.7). We summarized the Tukey-adjusted results using heatmaps of estimated marginal means for proportional abundance and richness across habitat types.

We tested the effect of environmental predictors on overall Bray-Curtis dissimilarity matrix using PERMANOVA, as explained in 2.5.1. We visualized results using boxplots, NMDS, hierarchical clustering, and density plots (see 2.5.1).

#### Quantification of Discriminant Taxa Across Habitats

To test our third hypothesis—bacterial communities exhibit uneven compositional differentiation across habitats—we quantified the degree to which community composition discriminates among habitats using complementary approaches that identify habitat-specific indicator taxa and evaluate classification performance. Habitats that act as distinct ecological endpoints host more distinct communities, whereas others support more overlapping assemblages, resulting in differences in discriminability.

We first identified bacterial taxa associated with specific habitats using indicator species analysis (IndVal) implemented in the R package *indicspecies*, v. 1.8.0 [54, 55]. The analysis was conducted on genus-level community matrices obtained by aggregating ASVs according to their taxonomic assignment (see 2.4). The IndVal statistic combines habitat specificity (relative abundance of a taxon in each habitat compared with others) and fidelity (frequency of occurrence within that habitat), producing values ranging from 0 (no association) to 1 (perfect association). Statistical significance was assessed using 9,999 permutations, with p-values adjusted using the false discovery rate (FDR). Genera with adjusted p-values < 0.1 were considered indicative of habitat association. For each indicator genus, we also calculated its prevalence (i.e., the proportion of samples in which the genus was detected within the associated habitat) and mean read abundance across samples in that habitat.

We then quantified how well bacterial community composition discriminates each habitat from all others using one-vs-rest random forest classifiers [56]. Read counts were aggregated at the family, genus, and species levels. The spring-fed pond habitat was excluded from this analysis because it was represented by only one sample. Models were fitted using class-balanced bootstrap sampling with 10,000 trees and evaluated using out-of-bag (OOB) accuracy and ROC AUC (Area Under the Receiver Operating Characteristic Curve). Analyses were repeated for four bacterial subsets: all taxa, potentially pathogenic, human-associated, and environmental bacteria.

## Results

### Description of Bacterial Community Composition and Richness Across Lanzarote Groundwater Ecosystems

**Fig. 2:**
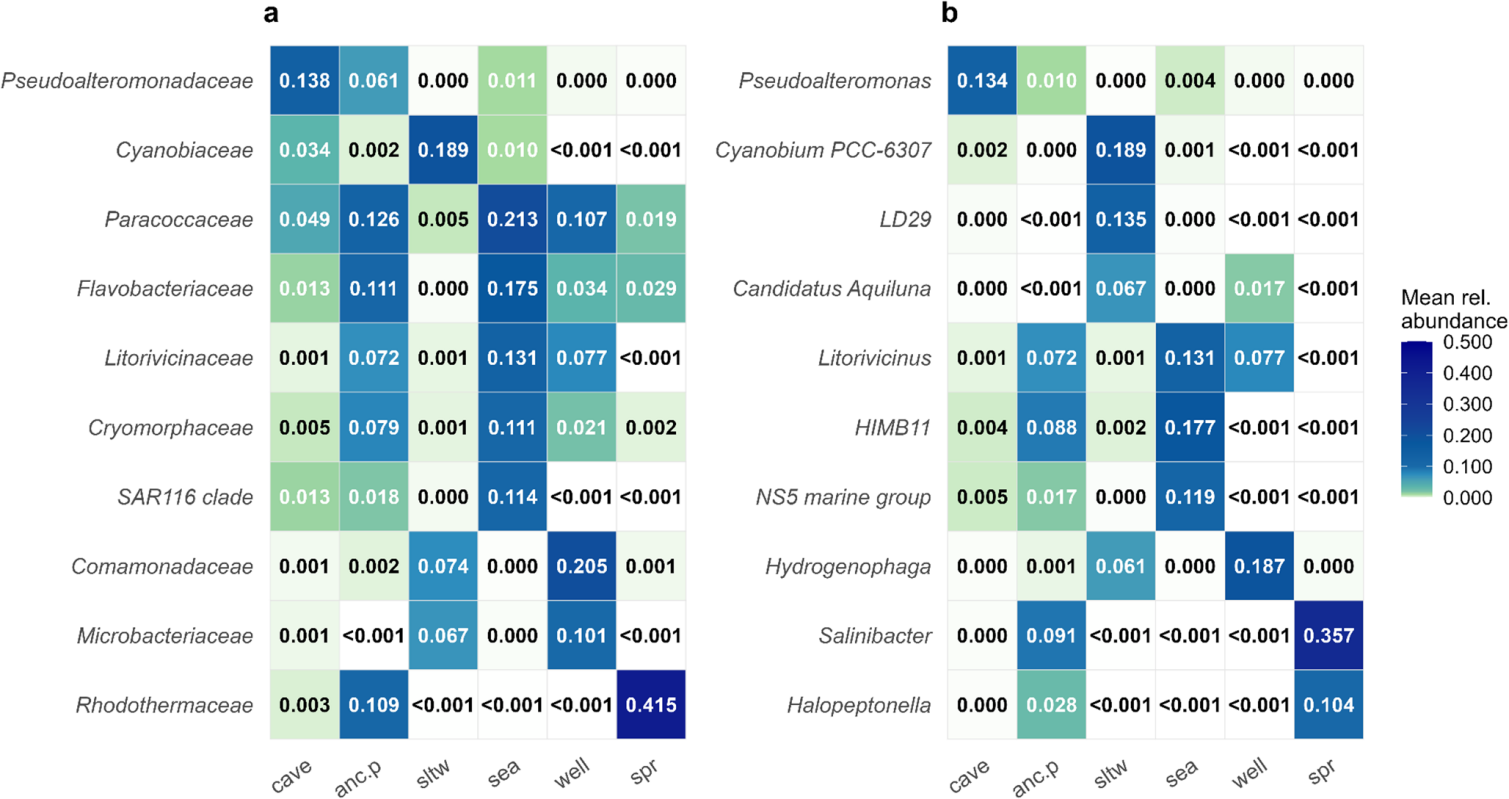
Composition of the bacterial community across habitat types. Heatmaps showing the mean relative abundance of the ten most abundant bacterial taxa across all samples. (**a**) Taxa classified at the family level. (**b**) Taxa classified at the genus level. Colours represent mean relative abundance within each habitat, and values inside the cells indicate the corresponding proportions. (cave = anchialine caves, anc.p = anchialine pools, sea = enclosed marine bays, sltw = saltwork pans, well = wells and water galleries, spr = spring-fed ponds)

Sequencing yielded a total of 2.72 million paired-end reads across the 28 samples. The number of raw reads per sample ranged from 1,226 to 142,323, with a mean of 90,748 ± 34,555 (mean ± standard deviation) reads per sample. We retrieved 2725 ASVs, of which 2241 could be taxonomically assigned to 248 bacterial families and 499 genera (Figs. 2 and 4). The remaining 484 ASVs lacked classification at the family or genus level but were assigned to higher taxonomic ranks (e.g., order, class, or phylum) (Table S4). The most abundant families, expressed as percentages of the total bacterial read count per sample, were Paracoccaceae (10.7%), Flavobacteriaceae (7.2%), Litorivicinaceae (5.8%), Pseudoalteromonadaceae (5.5%), and Cryomorphaceae (4.6%), followed by Comamonadaceae (4.3%), Rhodothermaceae (3.9%), and the SAR116 clade (3.2%, order Puniceispirillales). Paracoccaceae were most abundant in anchialine pools and coastal sites, while Comamonadaceae were more abundant in wells. Cyanobiaceae were primarily detected in saltworks’ saltpans whereas Pseudoalteromonadaceae were highly abundant in cave samples (Fig. 2a).

Dominant genera included HIMB11 (Rhodobacteraceae, 5.8%), *Litorivicinus* (5.8%), *Pseudoalteromonas* (4.2%), *Hydrogenophaga* (3.8%), and *Salinibacter* (3.2%) (Fig. 2b). HIMB11 was most abundant in anchialine pools and enclosed marine bays, *Hydrogenophaga* prevailed in wells, *Salinibacter* characterized the spring-fed pond, and *Pseudoalteromonas* dominated cave samples.

*Legionella*, *Vibrio*, *Psychrobacter*, and *Brevundimonas* were the most abundant of the 43 identified genera including potentially pathogenic bacteria (Figure S1a). *Vibrio* was particularly associated with anchialine pools and enclosed marine bays (“sea”, 1.5% each), whereas *Legionella* was the most abundant in the spring-fed pond site (0.4%) and in wells (0.71%). *Psychrobacter* reached its highest relative abundance in cave samples (3.0%), while *Brevundimonas* was among the most prevalent in saltworks’ saltpans (0.2%). Fecal-associated genera, such as *Enterococcus* and *Streptococcus*, were largely restricted to caves and wells but occurred at comparatively low relative abundances.

Human-related genera accounted for 5.5% of total bacterial abundance and were dominated by *Staphylococcus*, *Cutibacterium*, *Bacteroides*, *Brevibacterium*, and *Streptococcus* (Figure S1b). These genera were mostly detected in caves, anchialine pools, and wells, where they reached their highest relative abundance. In enclosed marine bays and saltworks sites, their relative abundance was consistently lower.

Dominant environmental taxa, namely those genera predominantly associated with natural environments rather than human-associated settings or potentially pathogenic lifestyles, included *Pseudoalteromonas*, *Litorivicinus*, *Hydrogenophaga*, *Cyanobium PCC-6307*, and *Salinibacter* (Figure S1c). These genera were very abundant in caves, anchialine pools, saltworks, and the spring-fed pond site, where *Salinibacter* and *Halopeptonella* together exceeded 45.0% relative abundance. Wells and enclosed marine bays were dominated by *Hydrogenophaga* and *Litorivicinus*, at 18.8% and 13.1% of total bacteria relative abundance, respectively.

**Fig. 3:**
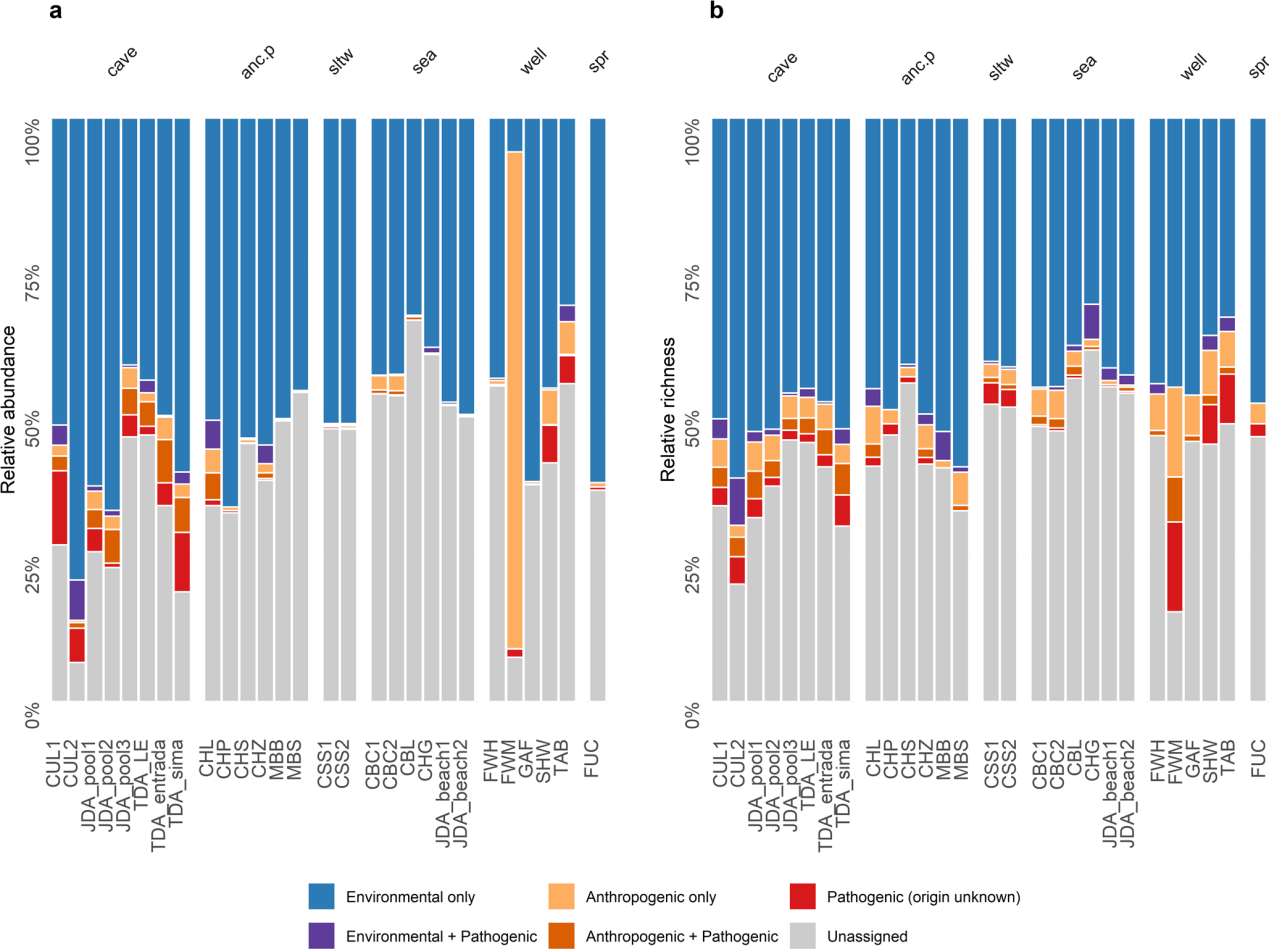
Functional composition of bacterial communities across the samples. (**a**) Relative abundance of functional categories calculated as the proportion of reads assigned to each category relative to the total bacterial community. (**b**) Relative richness expressed as the proportion of ASVs assigned to each category within each sample. Colors indicate functional categories based on trait assignments. (cave = anchialine caves, anc.p = anchialine pools, sea = enclosed marine bays, sltw = saltwork pans, well = wells and water galleries, spr = spring-fed ponds)

Environmental taxa dominated both the relative abundance and richness across most habitats, accounting for the majority of reads and ASVs in all samples (Fig. 3). Potentially pathogenic and human-related bacteria occurred across habitats but varied in relative abundance and richness among them. Their relative abundance and richness were consistently higher in caves and wells compared with coastal or hypersaline environments. These patterns motivated formal statistical tests to assess the significance and strength of habitat-associated differences.

### Inference of Habitat-Specific Patterns in the Bacterial Community

**Fig. 4:**
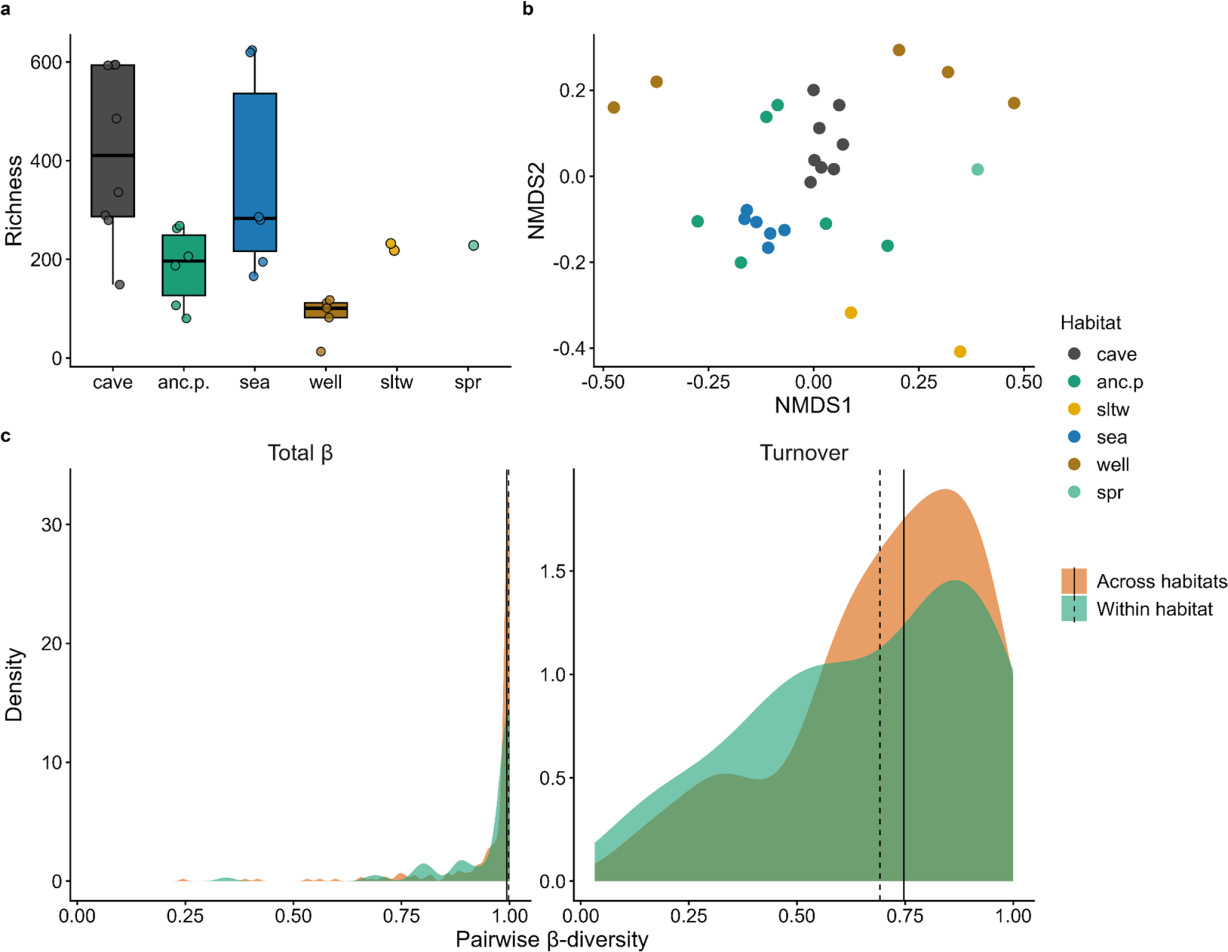
Whole-community bacterial patterns across habitat types. **(a)** Bacterial taxonomic (ASVs) richness across habitats. **(b)** NMDS ordination of community composition based on Bray–Curtis dissimilarities; points represent samples and are colored by habitat (stress = 0.177). **(c)** Density distributions of pairwise β-diversity values comparing within-habitat and across-habitat sample pairs for total β-diversity and its relative turnover component (β-turnover/β-total). The nestedness component is omitted because it represents the complement of turnover (β-nestedness/β-total = 1 − β-turnover/β-total). (cave = anchialine caves, anc.p = anchialine pools, sea = enclosed marine bays, sltw = saltwork pans, well = wells and water galleries, spr = spring-fed ponds)

Consistent with our first hypothesis, bacterial communities differed among habitats in both α- and β-diversity. α-diversity significantly differed across habitats, whether measured as taxonomic richness (GLM; χ² = 29.702, *p* < 0.001; Table 2; Fig. 4a) or Shannon diversity (LM, F = 3.656, *p* = 0.017; Table S6), independent of sequencing depth and other covariates. Pairwise comparisons for taxonomic richness showed significant differences between caves and anchialine pools (Tukey test; estimate = 0.816, *p* = 0.013; Table S5), caves and wells (estimate = 1.594, *p* < 0.0001), and enclosed marine bays and wells (estimate = 1.294, *p* = 0.006). Only caves and wells differed significantly when considering Shannon diversity (Tukey-adjusted *p* = 0.008; Table S7).

**Table 2:** Inference patterns of taxonomic richness of the total community as well as the proportion of potentially pathogenic, human-related, and environmental bacteria. Entire community richness was modeled using generalized linear models under a negative binomial distribution, whereas the proportion of reads assigned to potentially pathogenic, human-related, and environmental bacteria was modeled using a beta-binomial distribution. The models were fitted using richness as the response and habitat type, sequencing depth (reads), distance from the sea, and local building density (log(buildings + 1)) as predictors. (*Std* standard, *Df * degrees of freedom)

Bacterial community taxonomic richness						
	Estimate	Std. Error	z value	χ^2^	Df	*p* value
Habitat	NA	NA	NA	29.702	5	**< 0.0001**
Reads	1.72 × 10^− 6^	1.00 × 10^− 5^	0.171	0.028	1	0.865
Distance from sea	−5.94 × 10^− 6^	2.16 × 10^− 4^	−0.275	0.078	1	0.779
Nr. of buildings	0.057	0.069	0.931	0.816	1	0.366
Proportional abundance of potentially pathogenic bacteria (beta binomial)						
	**Estimate**	**Std. Error**	**z value**	**LR χ** ^**2**^	**Df**	**p value**
Habitat	NA	NA	NA	104.617	5	**< 0.0001**
Distance from sea	2.30 × 10^− 4^	2.40 × 10^− 4^	0.930	0.864	1	0.352
Nr. of buildings	0.177	0.078	2.254	5.078	1	**0.024**
Proportional abundance of human-related bacteria (beta binomial)						
	**Estimate**	**Std. Error**	**z value**	**LR χ** ^**2**^	**Df**	**p value**
Habitat	NA	NA	NA	6.791	5	0.236
Distance from sea	−0.004	0.003	−1.180	1.392	1	0.238
Nr. of buildings	0.153	0.119	1.278	1.632	1	0.201
Proportional abundance of environmental bacteria (beta binomial)						
	**Estimate**	**Std. Error**	**z value**	**LR χ** ^**2**^	**Df**	**p value**
Habitat	NA	NA	NA	18.495	5	**0.002**
Distance from sea	−3.00 × 10^− 4^	2.00 × 10^− 4^	−1.268	1.608	1	0.204
Nr. of buildings	−0.002	0.074	−0.02	0.004	1	0.983

Total β-diversity among samples was high, with a mean Bray–Curtis dissimilarity of 0.96 ± 0.10 (SD; *n* = 378 pairwise comparisons; see distribution in Fig. 4c), and 22% of its variance was explained by habitat type (PERMANOVA, *p* = 0.022; Table 3). Other covariates accounted for a negligible percentage of the variance (all R**²** < 0.04; *p* > 0.05), leaving 67% of the variance unexplained. Partitioning of β-diversity showed that turnover was the dominant component (mean β-turnover/β-total = 69%; mean β-nestedness/β-total = 31%; both expressed as proportions of total β-diversity; Fig. 4c), again mostly explained by habitat, although without statistical significance (Table 3). Hierarchical clustering produced patterns consistent with the NMDS ordination, supporting the distinct clustering of caves, hypersaline and saltwork habitats, while highlighting a greater degree of compositional overlap among wells, spring-fed ponds, and anchialine pools (Fig. 4; Figure S4).

**Table 3:** PERMANOVA results based on Bray–Curtis dissimilarities, showing the effects of habitat type and environmental variables on community composition, considering total β-diversity and its turnover and nestedness components across the whole community and functional groups. Reported values are R² from adonis2 models and represent the proportion of variation in compositional dissimilarity among samples explained by each predictor, not the magnitude of β-diversity itself. Negative R² values are shown as zero. Significant p-values (*p* < 0.05) are highlighted in bold

Subset	Predictor	Total		Nestedness		Turnover	
		*R*²	*p*-value	*R*²	*p*-value	*R*²	*p*-value
Whole community	Habitat	**0.218**	**0.022**	0.113	0.764	0.241	0.092
	Distance from sea	0.037	0.368	0.009	0.724	0.060	0.088
	Nr. of buildings	0.035	0.427	0.075	0.157	0.000	0.976
	Reads	0.039	0.261	0.080	0.133	1.198	0.373
	Residuals	0.670	–	0.721	–	0.667	–
Potentially pathogenetic bacteria	Habitat	**0.221**	**0.001**	0.161	0.612	0.321	0.129
	Distance from sea	0.037	0.305	0.057	0.232	0.000	0.903
	Nr. of buildings	0.035	0.541	0.018	0.693	0.036	0.510
	Reads	0.040	0.218	0.057	0.233	0.046	0.451
	Residuals	0.668	–	0.708	–	0.626	–
Human-associated	Habitat	**0.227**	**0.004**	0.265	0.133	0.206	0.391
	Distance from sea	0.039	0.207	0.022	0.597	0.076	0.208
	Nr. of buildings	0.033	0.628	0.002	0.990	0.114	0.065
	Reads	0.039	0.254	0.037	0.357	0.009	0.725
	Residuals	0.663	–	0.674	–	0.595	–
Environmental bacteria	Habitat	**0.213**	**0.040**	0.122	0.673	0.254	0.105
	Distance from sea	0.038	0.337	0.007	0.792	0.064	0.099
	Nr. of buildings	0.035	0.501	0.064	0.168	0.000	0.941
	Reads	0.038	0.312	**0.152**	**0.023**	0.009	0.889
	Residuals	0.676	–	0.655	–	0.677	–

Additional phylogenetic diversity analyses, including alternative metrics and model outputs, yielded consistent patterns across habitats and are reported in Supplementary Text 5 and Tables S15–S17. Furthermore, fine-scale analyses within La Corona lava tube further revealed consistent patterns across cave sections, with variation modulated by environmental gradients such as light regime and proximity to the sea (Supplementary Text 4).

### Patterns of Taxonomic Diversity of Bacterial Functional Groups

Consistent with our second hypothesis, potentially pathogenic and human-associated bacteria show habitat-specific enrichment and β-diversity dominated by nestedness, whereas the environmental fraction mirrors the whole community and remains turnover-dominated.

**Fig. 5:**
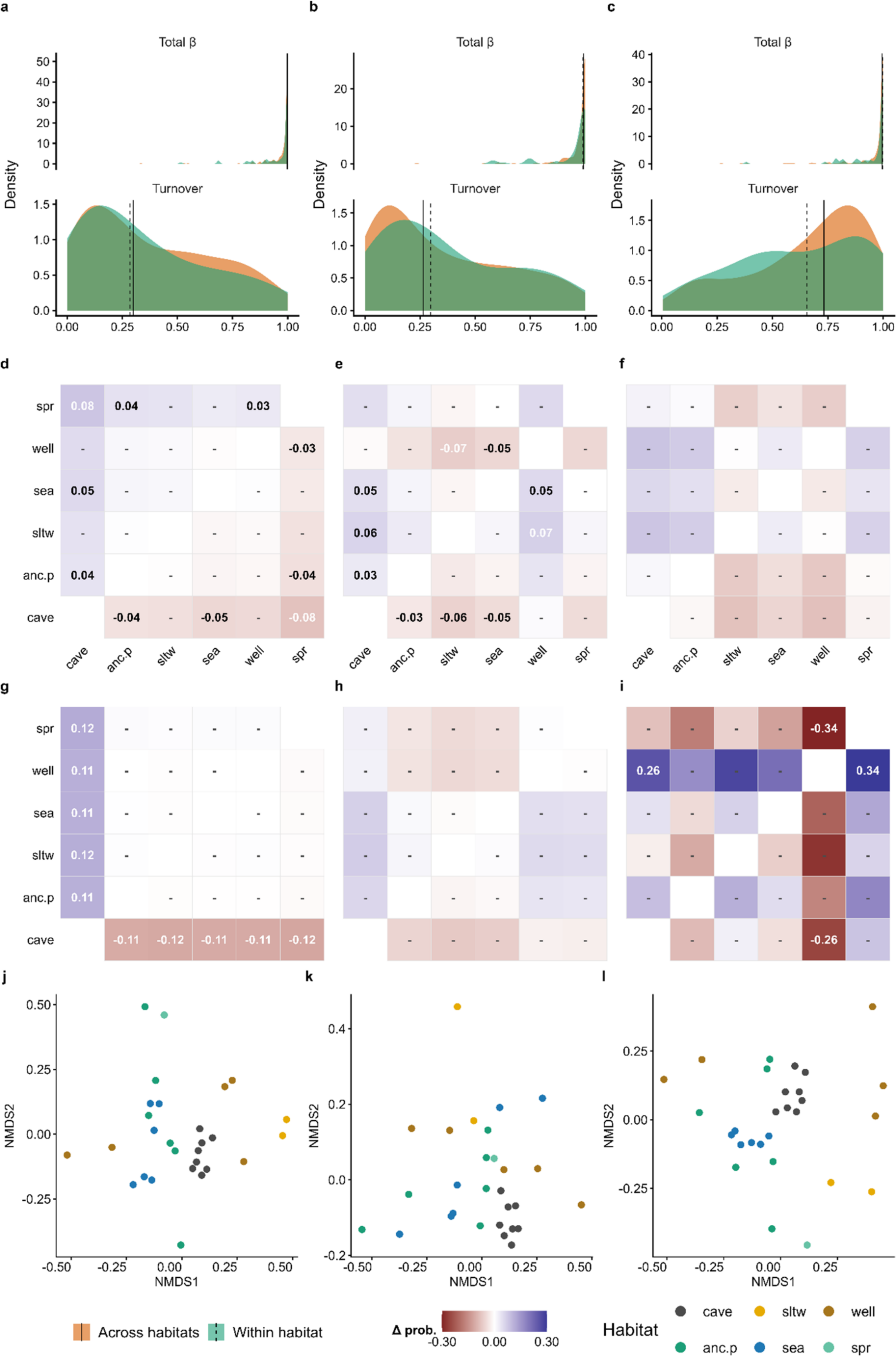
Comparative patterns of bacterial communities across habitat types. (**a–c**) Pairwise β-diversity distributions (β-total and percentage turnover components, β-turnover/β-total) comparing within-habitat versus across-habitat communities. The percentage of nestedness component is not shown, as it is the specular image of turnover (β-nestedness/β-total = 1 - β-turnover/β-total). (**d -f**) Pairwise differences in proportional richness of potentially pathogenic, human-related, and environmental bacteria across habitats. (**g-i**) Pairwise differences in proportional abundance for the same groups. Blue shades indicate higher values for the row compared to the column; red shades indicate lower values; non-significant contrasts are marked with “–”. (**j-l**) Non-metric multidimensional scaling (NMDS) ordinations of pathogenic, human-related and environmental bacteria, colored by habitat type. Stress values were 0.088 (j), 0.117 (k), and 0.169 (l). Legends are shared across rows: color scales indicate the range of pairwise differences; shaded density areas show β-diversity comparisons (green = within habitats, orange = across habitats); NMDS colours correspond to habitat types (cave = anchialine caves, anc.p = anchialine pools, sea = enclosed marine bays, sltw = saltwork pans, well = wells and water galleries, spr = spring-fed ponds)

Both proportional abundance and richness of potentially pathogenic bacteria were significantly affected by habitat type (GLM, χ² = 104.617, *p* < 0.0001 Table 2) and the number of buildings within a 1-km radius (GLM, estimate = 0.177, *p* = 0.02; Fig. 5g; Table 2). Pairwise comparisons indicated that this pattern was driven by higher proportional abundance of pathogens in caves compared to other habitats (Table S10; Fig. 5g). Their proportional richness was also significantly affected by habitat type (GLM, χ² = 59.890, *p* < 0.0001; Fig. 5d; Table S9), but also by the number of reads, although the effect size was negligible (estimate ≈ 0.000, *p* = 0.039; Table S9). Composition of potentially pathogenic bacteria differed strongly among samples, with nestedness contributing more strongly than turnover to the observed β-diversity patterns (β-nestedness/β-total = 0.62 ± 0.28; Fig. 5a). Differences in β-total were attributed to habitats (PERMANOVA, R² = 0.22, *p* = 0.001) (Fig. 5a, j; Table 3), although most of the β-total variance remained unexplained (residuals R² = 0.66).

Proportional abundance of potentially human-associated bacteria was not significantly affected by any predictor (Fig. 5h; Table 3, S9); whereas their proportional richness was significantly influenced by habitat type (GLM, χ² = 33.924, *p* < 0.0001) mostly due to the enrichment of caves and wells (Fig. 5e, Tables S9–S10). Their community composition was primarily driven by nestedness (β-nestedness/β-total = 0.64 ± 0.29; Fig. 5b, k). Most of the variance in β-total was explained by habitat (PERMANOVA, R² = 0.23, *p* = 0.04), although most of the β-total variance remained unexplained (residuals R² = 0.66) (Table 3).

Proportional abundance of environmental bacteria was significantly influenced by habitat type (GLM, χ² = 18.495, *p* < 0.0001; Table 2), due to differences amongst caves, wells, and springs (Fig. 5i; Table S8). Their proportional richness was significantly affected both by habitat (χ² = 15.574, *p* = 0.008; Fig. 5f; Table S9) and negatively by the number of buildings (Estimate = −0.048, *p* = 0.013). In contrast to potentially pathogenic and human-related bacteria, community composition of environmental bacteria was primarily driven by turnover (β-turnover/β-total = 0.65 ± 0.27; Fig. 5c, l). Most of the variance in β-total was explained by habitat (PERMANOVA, R² = 0.213, *p* = 0.04), although most of the β-total variance remained unexplained (residuals R² = 0.68) (Table 3).

### Habitat-associated and Discriminant Bacterial Taxa

Consistent with hypothesis three, bacterial communities exhibited uneven compositional differentiation across habitats. This pattern was supported by indicator species analysis and random forest classification. These analyses identified the taxa underlying the patterns described above.

Indicator species analysis identified taxa with strong habitat-specific associations (see Sect. 3.1) for anchialine caves and saltwater environments (Table S11). Several genera exhibited very high indicator values (IndVal ≥ 0.95) and near-complete prevalence, indicating strong habitat fidelity (e.g. *Idiomarina*, *Marinobacter*), together with taxa typically associated with host-related or human-impacted environments (e.g. *Cutibacterium*, *Streptococcus*). Saltwater habitats, including both coastal seawater and saltwork pans, also displayed highly consistent indicator taxa despite the relatively small number of samples. Representative examples include *Cyanobium* (saltwork pans) and members of the NS5 marine group and *Amylibacter* (coastal seawater), all showing high indicator values (IndVal ≥ 0.9) and full prevalence. In contrast, anchialine pools and wells showed weaker and less consistent associations. No genera remained significant after strict correction for multiple testing (FDR < 0.05), although several taxa reached significance under a relaxed threshold (FDR < 0.1). These taxa displayed intermediate indicator values and moderate prevalence, suggesting weaker habitat specificity and greater community overlap.

These patterns were mirrored by the results of the random forest analyses, which quantify how well the habitat can be predicted from the community composition. Saltworks, enclosed marine bays, and anchialine caves showed the highest and most consistent predictability across taxonomic ranks and data subsets. Saltworks and enclosed marine bays achieved ROC AUC values generally exceeding 0.90 and out-of-bag (OOB) accuracies above 0.85, while anchialine caves also showed strong performance (AUC typically 0.80–0.90; Figure S5).

By contrast, anchialine pools and wells were less predictable (AUC often < 0.75), consistent with their weaker indicator signals. Discriminability was generally highest at the family level, followed by genus, and declined at species level, where classification frequently failed. Across community subsets, models based on the full bacterial community consistently outperformed those based on functional subsets, while environmentally associated taxa showed intermediate performance. Models restricted to potentially pathogenic and human-associated bacteria showed reduced predictability overall, except in anchialine caves and saltworks, where performance remained comparatively high (Figure S5).

## Discussion

### Groundwater Ecosystems in Lanzarote are Reservoirs of Human-associated and Pathogenic Bacteria

Overall, our results support the proposed framework of community assembly. Patterns of β-diversity in the whole community are consistent with turnover-dominated structuring driven by environmental filtering, whereas functional subsets reveal nestedness-dominated patterns associated with anthropogenic enrichment. Differences in discriminability across habitats further confirm uneven compositional differentiation, reflecting variation in the strength of environmental and anthropogenic drivers.

Groundwater-dependent ecosystems in Lanzarote support distinct bacterial assemblages but also contain human-derived and clinically relevant bacteria. Detection of genera linked to fecal contamination suggests potential impacts on the integrity of the island’s aquifer and its capacity to buffer human-derived pollutants. While our sampling design does not pinpoint the specific sources of contamination, the association between habitat type and the enrichment of potentially pathogenic bacteria suggests that compartmentalized systems with high residence time may reflect anthropogenic pressure.

Cave sites were relatively enriched in clinically relevant taxa such as *Streptococcus*, *Psychrobacter*, and *Mycobacterium*, which together accounted for a notable proportion of potentially pathogenic bacteria relative abundance (up to 27%). We also detected fecal-associated genera (e.g., *Bacteroides*, *Enterococcus*) and human-associated taxa such as *Staphylococcus* and *Cutibacterium* [57–59]. These findings suggest that lava tubes may act as accumulation zones of externally introduced microorganisms. Their limited water renewal likely reduces microbial flushing, allowing contaminants to accumulate rather than solely reflecting natural community composition. This interpretation is consistent with previous detections of fecal bacteria linked to tourist activity in the Jameos del Agua [60]. Although anthropogenic inputs dominate in impacted caves, natural gradients (e.g., salinity, stratification) can also structure microbial communities, as shown in pristine anchialine systems [61]. Fine-scale cave-specific analyses further suggest that pathogen and human-associated bacterial enrichment is consistent across cave subsites and is modulated by light regime and proximity to the sea (Supplementary Text 4).

In contrast, enclosed marine bays and hypersaline habitats were dominated by environmental specialists (e.g., *Pseudoalteromonas*, *Litorivicinus*, *Cyanobium*), consistent with strong environmental filtering along physicochemical gradients. This pattern is consistent with the coexistence of environmental filtering in open systems and the accumulation of human-associated taxa in more isolated habitats.

Notably, dominant taxa were not the main drivers of habitat discrimination in the inferential analyses. Taxa such as *Cyanobium* in saltwork pans or members of the NS5 marine group in coastal seawater exhibited high indicator values and near-complete habitat fidelity, making them strong discriminators. Similarly, in anchialine caves, genera such as *Idiomarina* and *Marinobacter* combined high prevalence with strong specificity, whereas other abundant taxa were more widely distributed across habitats. Thus, specialized taxa better capture ecological differences among habitats [62]. Such discrepancies are expected in microbial systems, where community structure is shaped by both ubiquitous generalists and habitat-restricted specialists.

### Turnover Dominates Whole Bacterial β-diversity Across Habitats

Our results demonstrate that bacterial communities in the groundwater-dependent habitats of Lanzarote differ in taxonomic composition, with species turnover representing the dominant component of β-diversity. Habitat type contributed significantly to community differences. This pattern is consistent with partial environmental filtering acting across the studied habitats, although the substantial overlap among communities suggests that dispersal and hydrological connectivity also contribute to community assembly [63–65]. Similar patterns have been reported in coastal aquifers, where salinity gradients act as major environmental filters structuring microbial communities [66–68].

Phylogenetic analyses further showed that habitat type constrains the evolutionary breadth of bacterial communities. Caves supported higher taxonomic and phylogenetic diversity, consistent with their role as refugia for diverse and distinct lineages, whereas wells exhibited reduced diversity, likely reflecting limited resource availability. These results suggest that environmental filtering operates not only at the taxonomic level but also constrains phylogenetic diversity, limiting the range of lineages present [69–71]. Together, these findings reinforce the role of both ecological sorting and evolutionary filtering across habitats, while phylogenetic nestedness reflects evolutionary filtering of persistent lineages [72].

From a conservation perspective, these results highlight anchialine caves as both reservoirs of phylogenetically distinct bacterial lineages and sinks of anthropogenic pollution. Integrating microbial indicators into groundwater monitoring could therefore provide an early-warning framework for ecosystem degradation and contamination.

### Nestedness Dominates Human-associated Lineages, Indicating Punctual Enrichment

Functional group analyses suggested that human-related and potentially pathogenic bacteria exhibit diversity patterns dominated by nestedness, contrasting with the turnover-driven structure of the overall bacterial community. Anthropogenic inputs and subsurface hydrological connectivity likely facilitate the redistribution of human-associated bacteria across groundwater habitats [73, 74].

The observation that human-related taxa are enriched in caves but not exclusively confined to them reflects a broader pattern of diffuse bacterial dispersal through coastal aquifers. Rather than being restricted to point-source contamination sites, human-derived bacteria appear capable of spreading across multiple groundwater-fed habitats, likely facilitated by subsurface hydrological connectivity. Their persistence likely depends on hydrodynamics such as residence time and flushing rates [75]. However, their presence does not necessarily imply direct human introduction, as some taxa may also be transported by environmental or wildlife-mediated pathways.

It remains unclear whether these taxa represent stable residents or transient inputs. While some fecal indicator bacteria may persist under oligotrophic and dark conditions [76], others likely reflect episodic inputs driven by recharge events or human activity [77, 78]. Similar temporal variability has been reported in anchialine systems, where pathobiome signals often fluctuate in response to seasonal or anthropogenic pulses rather than stable community membership [79]. This enrichment has implications for both ecosystem functioning and public health. These shifts may introduce pathogens of anthropogenic origin [50] and alter trophic interactions, potentially affecting endemic fauna and food web structure [80–82].

### The Distinctiveness of Caves

Cave environments emerged as highly distinctive bacterial habitats in our dataset, consistently showing high discriminability in random forest models across taxonomic ranks and functional groups. In our study, cave-associated bacterial communities differed markedly from those in wells, spring-fed ponds, and anchialine pools (Figs. 3 and 4), underscoring caves as distinct ecological units shaped by unique environmental conditions and limited hydrological connectivity. This pattern persists even in hydrologically dynamic systems, where tidal forcing does not necessarily translate into strong mixing between marine and groundwater masses [39].

Similar degrees of community differentiation have been widely reported in anchialine and aquatic cave systems, where microbial assemblages are strongly structured by salinity, oxygen, and organic matter gradients, and often display high spatial heterogeneity even across nearby sites [20, 21, 83–88].

However, Lanzarote caves also accumulate human-derived bacteria, pointing to aquifer contamination likely driven by tourism infrastructure and historical groundwater exploitation [89]. This suggests that while ecological drivers are consistent, the degree of anthropogenic influence varies among systems. Overall, our results reinforce the ecological distinctiveness of cave habitats as reservoirs of bacterial diversity.

Saltworks also emerged as highly distinctive habitats, where extreme salinity (~ 95‰) selects for specialized halophilic taxa and excludes generalist and anthropogenic bacteria [90]. These systems exhibited high turnover, low nestedness, and strong predictability in random forest models, consistent with strong environmental filtering.

By contrast, anchialine pools were among the least distinct habitats, consistent with their role as ecotonal systems influenced by both marine and terrestrial inputs. These environments showed greater taxonomic overlap with other habitats, lower classification accuracy, and intermediate diversity patterns, reflecting diffuse environmental filtering and higher connectivity.

### Towards Effective Protection of Coastal Aquifers Considering Hydrological and Biological Components

These findings, particularly the contrasting roles of environmental filtering and anthropogenic enrichment, have direct implications for groundwater management. Groundwater-dependent habitats in Lanzarote support diverse ecosystem services, including freshwater provision, salt production, fisheries, coastal productivity, and cultural value. However, this multifunctionality is coupled with pronounced ecological fragility. These systems rely on tightly coupled geological, hydrological, and biological interactions that can be disrupted by contamination or hydrological change. Even minimal anthropogenic inputs may trigger cascading effects across microbial and faunal networks, ultimately degrading both ecosystem integrity and water quality (Table S1).

The detection of human-derived and clinically relevant bacteria (e.g., *Enterococcus*, *Streptococcus*) highlights that aquifer systems can act both as reservoirs of biodiversity and as sinks of contamination, with potential public health implications. From a management perspective, caves and wells should be treated as sentinel habitats for monitoring human pressures on groundwater. Integrating microbial diversity and pathobiome assessments into water-quality programs could provide early-warning indicators of aquifer degradation. To ensure comparability, we recommend standardized markers (e.g., 16S rRNA V3–V4) and harmonized protocols for DNA extraction, amplification, and analysis. Future studies should incorporate temporal variability (e.g., seasonal sampling) to better capture shifts in microbial communities.

Current groundwater management frameworks, including the European Water Framework Directive, largely overlook biological indicators, limiting the assessment of subterranean ecosystems [91–93]. In the Canary Islands, management plans focus primarily on quantitative and chemical parameters, with limited consideration for biological processes or groundwater–coastal connectivity. In addition, key habitats such as anchialine systems and submarine groundwater discharge zones remain incompletely mapped and monitored [94]. These gaps highlight the need to integrate biological indicators into existing monitoring frameworks through a bio-hydrological approach.

By integrating hydrological, chemical, and biological monitoring, Lanzarote could serve as a model for ecosystem-based groundwater governance in outermost EU regions. Such integration is essential to safeguard subterranean biodiversity, maintain ecosystem services, and ensure the long-term sustainability of groundwater resources.

## Supplementary Information

Below is the link to the electronic supplementary material.

Supplementary Material 1 (XLSX 885 KB)

Supplementary Material 2 (DOCX 4.06 MB)

## Data Availability

The sequencing data have been deposited in NCBI Sequence Read Archive (SRA) under BioProject accession number PRJNA1390142.The R scripts for the DADA2 pipeline and the statistical analyses and figure generation are publicly available on GitHub at https://github.com/francescodinezio/lanzarote-16 S-pipeline.git and https://github.com/francescodinezio/lanzarote-groundwater-microbial-ecology.git; as well as on Zenodo (DOI: 10.5281/zenodo.19483763).

## References

[1] Gillespie RG, Clague DA (2009) Encyclopedia of Islands. University of California Press

[2] Tershy BR, Shen K-W, Newton KM et al (2015) The importance of islands for the protection of biological and linguistic diversity. BioScience 65:592–597. 10.1093/biosci/biv031

[3] Fernández-Palacios JM, Kreft H, Irl SDH et al (2021) Scientists’ warning - the outstanding biodiversity of islands is in peril. Glob Ecol Conserv 31:e01847. 10.1016/j.gecco.2021.e01847 34761079 10.1016/j.gecco.2021.e01847 PMC8556160

[4] Moore WS (2010) The effect of submarine groundwater discharge on the ocean. Ann Rev Mar Sci 2:59–88. 10.1146/annurev-marine-120308-081019 21141658 10.1146/annurev-marine-120308-081019

[5] Moosdorf N, Stieglitz T, Waska H et al (2015) Submarine groundwater discharge from tropical islands: a review. Grundwasser 20:53–67. 10.1007/s00767-014-0275-3

[6] Luijendijk E, Gleeson T, Moosdorf N (2020) Fresh groundwater discharge insignificant for the world’s oceans but important for coastal ecosystems. Nat Commun 11:1260. 10.1038/s41467-020-15064-8 32152309 10.1038/s41467-020-15064-8 PMC7062736

[7] Gibson VL, Bremer LL, Burnett KM et al (2022) Biocultural values of groundwater dependent ecosystems in Kona, Hawaiʻi. Ecol Soc 27. 10.5751/ES-13432-270318

[8] Mammola S, Brankovits D, Lorenzo TD et al (2026) Subterranean environments contribute to three-quarters of classified ecosystem services. Biol Rev. 10.1002/brv.70137 41665280 10.1002/brv.70137 PMC13149732

[9] Moore WS (1999) The subterranean estuary: a reaction zone of ground water and sea water. Mar Chem 65:111–125. 10.1016/S0304-4203(99)00014-6

[10] Santos IR, Chen X, Lecher AL et al (2021) Submarine groundwater discharge impacts on coastal nutrient biogeochemistry. Nat Rev Earth Environ 2:307–323. 10.1038/s43017-021-00152-0

[11] Jang C-S, Liu C-W, Chen S-K, Lin W-S (2012) Using a Mass Balance Model to Evaluate Groundwater Budget of Seawater-Intruded Island Aquifers. JAWRA J Am Water Resour Association 48:61–73. 10.1111/j.1752-1688.2011.00593.x

[12] Cho H-M, Kim G, Kwon EY et al (2018) Radium tracing nutrient inputs through submarine groundwater discharge in the global ocean. Sci Rep 8:2439. 10.1038/s41598-018-20806-2 29403050 10.1038/s41598-018-20806-2 PMC5799265

[13] Kim KH, Michael HA, Field EK, Ullman WJ (2019) Hydrologic shifts create complex transient distributions of particulate organic carbon and biogeochemical responses in beach aquifers. J Geophys Res Biogeosci 124:3024–3038. 10.1029/2019JG005114

[14] Moore WS, Joye SB (2021) Saltwater Intrusion and Submarine Groundwater Discharge: Acceleration of Biogeochemical Reactions in Changing Coastal Aquifers. Front Earth Sci 9. 10.3389/feart.2021.600710

[15] Brankovits D, Pohlman JW, Niemann H et al (2017) Methane- and dissolved organic carbon-fueled microbial loop supports a tropical subterranean estuary ecosystem. Nat Commun 8:1835. 10.1038/s41467-017-01776-x 29180666 10.1038/s41467-017-01776-x PMC5703975

[16] Ruiz-González C, Rodellas V, Garcia-Orellana J (2021) The microbial dimension of submarine groundwater discharge: current challenges and future directions. FEMS Microbiol Rev 45:fuab010. 10.1093/femsre/fuab010 33538813 10.1093/femsre/fuab010 PMC8498565

[17] Elbourne LDH, Sutcliffe B, Humphreys W et al (2022) Unravelling Stratified Microbial Assemblages in Australia’s Only Deep Anchialine System, The Bundera Sinkhole. Front Mar Sci 9. 10.3389/fmars.2022.872082

[18] Narasingarao P, Podell S, Ugalde JA et al (2012) *De novo* metagenomic assembly reveals abundant novel major lineage of Archaea in hypersaline microbial communities. ISME J 6:81–93. 10.1038/ismej.2011.78 21716304 10.1038/ismej.2011.78 PMC3246234

[19] Vaidya S, Dev K, Sourirajan A (2018) Distinct Osmoadaptation Strategies in the Strict Halophilic and Halotolerant Bacteria Isolated from Lunsu Salt Water Body of North West Himalayas. Curr Microbiol 75:888–895. 10.1007/s00284-018-1462-8 29480323 10.1007/s00284-018-1462-8

[20] Pohlman JW (2011) The biogeochemistry of anchialine caves: progress and possibilities. Hydrobiologia 677:33–51. 10.1007/s10750-011-0624-5

[21] Kajan K, Cukrov N, Cukrov N et al (2022) Microeukaryotic and prokaryotic diversity of anchialine caves from Eastern Adriatic Sea Islands. Microb Ecol 83:257–270. 10.1007/s00248-021-01760-5 33903927 10.1007/s00248-021-01760-5 PMC8891109

[22] Meland K, Iliffe TM, Thiele S (2023) A glimpse into the dark – the bacterial and archaeal diversity of tropical anchialine cave sediments. Access Microbiology 5:000622.v3. 10.1099/acmi.0.000622.v3

[23] Kochary S, Alnaqshabandi L, Abdo H (2018) The impact of residential septic tanks on soil water infiltration and groundwater contamination in Duhok city. Acad J Nawroz Univ 7:245. 10.25007/ajnu.v7n4a297

[24] Dey U, Sarkar S, Duttagupta S et al (2022) Influence of Hydrology and Sanitation on Groundwater Coliform Contamination in Some Parts of Western Bengal Basin: Implication to Safe Drinking Water. Front Water 4. 10.3389/frwa.2022.875624

[25] Dong Y, Jiang Z, Hu Y et al (2024) Pathogen contamination of groundwater systems and health risks. Critical Reviews in Environmental Science and Technology 54:267–289. 10.1080/10643389.2023.2236486

[26] Song S-H, Zemansky G (2012) Vulnerability of groundwater systems with sea level rise in coastal aquifers, South Korea. Environ Earth Sci 65:1865–1876. 10.1007/s12665-011-1169-7

[27] Oberle FKJ, Swarzenski PW, Storlazzi CD (2017) Atoll Groundwater Movement and Its Response to Climatic and Sea-Level Fluctuations. Water 9:650. 10.3390/w9090650

[28] Benz SA, Irvine DJ, Rau GC et al (2024) Global groundwater warming due to climate change. Nat Geosci 17:545–551. 10.1038/s41561-024-01453-x

[29] Cuthbert MO, Gleeson T, Moosdorf N et al (2019) Global patterns and dynamics of climate–groundwater interactions. Nature Clim Change 9:137–141. 10.1038/s41558-018-0386-4

[30] Fatima K (2024) Exploring groundwater–climate change interactions: A critical review of Groundwater and Climate Change: Multi-Level Law and Policy Perspectives by Philippe Cullet. World Water Policy 10:62–70. 10.1002/wwp2.12142

[31] Carlson CJ, Albery GF, Merow C et al (2022) Climate change increases cross-species viral transmission risk. Nature 607:555–562. 10.1038/s41586-022-04788-w 35483403 10.1038/s41586-022-04788-w

[32] Mora C, McKenzie T, Gaw IM et al (2022) Over half of known human pathogenic diseases can be aggravated by climate change. Nat Clim Chang 12:869–875. 10.1038/s41558-022-01426-1 35968032 10.1038/s41558-022-01426-1 PMC9362357

[33] He Z, Zhang P, Wu L et al (2018) Microbial functional gene diversity predicts groundwater contamination and ecosystem functioning. mBio 9:10.1128/mbio.02435-17. 10.1128/mbio.02435-17 10.1128/mBio.02435-17 PMC5821090 29463661

[34] Lyons KJ, Hokajärvi A-M, Ikonen J et al (2021) Surface water intrusion, land use impacts, and bacterial community composition in shallow groundwater wells supplying potable water in sparsely populated areas of a boreal region. Microbiology Spectrum 9:e00179-21. 10.1128/Spectrum.00179-21 34730413 10.1128/Spectrum.00179-21 PMC8567237

[35] Ferguson G, Gleeson T (2012) Vulnerability of coastal aquifers to groundwater use and climate change. Nat Clim Change 2:342–345. 10.1038/nclimate1413

[36] Martinez A, Gonzalez BC, Núñez J (2016) Guide to the anchialine ecosystems of Los Jameos del Agua and Túnel de la Atlántida. Cabildo de Lanzarote

[37] Martínez A, García-Gómez G, García-Herrero Á (2019) Lanzarote and Chinijo Islands: An Anchialine UNESCO Global Geopark. In: Mateo E, Martínez-Frías J, Vegas J (eds) Lanzarote and Chinijo Islands Geopark: From Earth to Space. Springer International Publishing, Cham, pp 109–121

[38] Herlemann DPR, Labrenz M, Jürgens K et al (2011) Transitions in bacterial communities along the 2000 km salinity gradient of the Baltic Sea. The ISME Journal 5:1571–1579. 10.1038/ismej.2011.41 21472016 10.1038/ismej.2011.41 PMC3176514

[39] Wilkens H, Iliffe TM, Oromí P et al (2009) The Corona lava tube, Lanzarote: geology, habitat diversity and biogeography. Mar Biodiv 39:155–167. 10.1007/s12526-009-0019-2

[40] Callahan BJ, McMurdie PJ, Rosen MJ et al (2016) DADA2: High-resolution sample inference from Illumina amplicon data. Nat Methods 13:581–583. 10.1038/nmeth.3869 27214047 10.1038/nmeth.3869 PMC4927377

[41] R Core Team (2025) R: A Language and. Environment for Statistical Computing

[42] Göker M, Christensen H, Fingerle V et al (2025) List of recommended names for bacteria of medical importance: report of the Ad Hoc Committee on Mitigating Changes in Prokaryotic Nomenclature. Int J Syst Evol Microbiol 75:006943. 10.1099/ijsem.0.006943 41129200 10.1099/ijsem.0.006943 PMC12548757

[43] Freese HM, Meier-Kolthoff JP, Sardà Carbasse J et al (2026) TYGS and LPSN in 2025: a global core biodata resource for genome-based classification and nomenclature of prokaryotes within DSMZ Digital Diversity. Nucleic Acids Res 54:D884–D891. 10.1093/nar/gkaf1110 41160892 10.1093/nar/gkaf1110 PMC12807606

[44] Oksanen J, Simpson GL, Blanchet FG et al (2025) vegan: Community Ecology Package

[45] Cardoso P, Rigal F, Carvalho JC (2015) BAT – Biodiversity Assessment Tools, an R package for the measurement and estimation of alpha and beta taxon, phylogenetic and functional diversity. Methods Ecol Evol 6:232–236. 10.1111/2041-210X.12310

[46] Venables WN, Ripley BD (2002) Modern Applied Statistics with S, Fourth. Springer US

[47] Lüdecke D, Ben-Shachar MS, Patil I et al (2021) Performance: an R package for assessment, comparison and testing of statistical models. J Open Source Softw 6:3139. 10.21105/joss.03139

[48] Zuur AF, Ieno EN (2016) A protocol for conducting and presenting results of regression-type analyses. Methods Ecol Evol 7:636–645. 10.1111/2041-210X.12577

[49] Fox J, Weisberg S (2018) An R companion to applied regression. Sage publications

[50] Lenth RV (2025) emmeans: Estimated Marginal Means, aka Least-Squares Means. 10.32614/CRAN.package.emmeans

[51] Wickham H (2016) ggplot2: Elegant Graphics for Data Analysis, 2nd ed. Springer, Cham

[52] R Core Team (2023) R: A Language and. Environment for Statistical Computing

[53] Brooks ME, Kristensen K, Benthem KJ et al (2017) glmmTMB balances speed and flexibility among packages for zero-inflated generalized linear mixed modeling. R J 9:378. 10.32614/RJ-2017-066

[54] Dufrêne M, Legendre P (1997) Species assemblages and indicator species:the need for a flexible asymmetrical approach. Ecol Monogr 67:345–366. 10.1890/0012-9615(1997)067[0345:SAAIST]2.0.CO;2

[55] De Cáceres M, Legendre P (2009) Associations between species and groups of sites: indices and statistical inference. Ecology 90:3566–3574. 10.1890/08-1823.1 20120823 10.1890/08-1823.1

[56] Brieuc MSO, Waters CD, Drinan DP, Naish KA (2018) A practical introduction to Random Forest for genetic association studies in ecology and evolution. Mol Ecol Resour 18:755–766. 10.1111/1755-0998.12773 29504715 10.1111/1755-0998.12773

[57] Sinton LW, Donnison AM, Hastie CM (1993) Faecal streptococci as faecal pollution indicators: A review. Part II: Sanitary significance, survival, and use. N Z J Mar Freshwat Res 27:117–137. 10.1080/00288330.1993.9516550

[58] Teixeira P, Dias D, Costa S et al (2020) *Bacteroides* spp. and traditional fecal indicator bacteria in water quality assessment – an integrated approach for hydric resources management in urban centers. J Environ Manage 271:110989. 10.1016/j.jenvman.2020.110989 32579514 10.1016/j.jenvman.2020.110989

[59] Li E, Saleem F, Edge TA, Schellhorn HE (2021) Biological indicators for fecal pollution detection and source tracking: a review. Processes 9:2058. 10.3390/pr9112058

[60] Martínez A, Di Cesare A, Mari-Mena N et al (2020) Tossed ‘good luck’ coins as vectors for anthropogenic pollution into aquatic environment. Environ Pollut 259:113800. 10.1016/j.envpol.2019.113800 31887589 10.1016/j.envpol.2019.113800

[61] Vojvoda Zeljko T, Kajan K, Jalžić B et al (2024) Genome-centric metagenomes unveiling the hidden resistome in an anchialine cave. Environ Microbiome 19:67. 10.1186/s40793-024-00612-2 39252078 10.1186/s40793-024-00612-2 PMC11386340

[62] Eckert EM, Amalfitano S, Di Cesare A et al (2020) Different substrates within a lake harbour connected but specialised microbial communities. Hydrobiologia 847:1689–1704. 10.1007/s10750-019-04068-1

[63] Stegen JC, Fredrickson JK, Wilkins MJ et al (2016) Groundwater–surface water mixing shifts ecological assembly processes and stimulates organic carbon turnover. Nat Commun 7:11237. 10.1038/ncomms11237 27052662 10.1038/ncomms11237 PMC4829693

[64] Christensen GA, Moon J, Veach AM et al (2018) Use of in-field bioreactors demonstrate groundwater filtration influences planktonic bacterial community assembly, but not biofilm composition. PLoS One 13:e0194663. 10.1371/journal.pone.0194663 29558522 10.1371/journal.pone.0194663 PMC5860781

[65] Danczak RE, Johnston MD, Kenah C et al (2018) Microbial community cohesion mediates community turnover in unperturbed aquifers. mSystems 3:10.1128/msystems.00066 − 18. 10.1128/msystems.00066-18 10.1128/mSystems.00066-18 PMC6030547 29984314

[66] Lee E, Shin D, Hyun SP et al (2017) Periodic change in coastal microbial community structure associated with submarine groundwater discharge and tidal fluctuation. Limnol Oceanogr 62:437–451. 10.1002/lno.10433

[67] Héry M, Volant A, Garing C et al (2014) Diversity and geochemical structuring of bacterial communities along a salinity gradient in a carbonate aquifer subject to seawater intrusion. FEMS Microbiol Ecol 90:922–934. 10.1111/1574-6941.12445 25348057 10.1111/1574-6941.12445

[68] Adyasari D, Hassenrück C, Montiel D, Dimova N (2020) Microbial community composition across a coastal hydrological system affected by submarine groundwater discharge (SGD). PLoS One 15:e0235235. 10.1371/journal.pone.0235235 32598345 10.1371/journal.pone.0235235 PMC7323985

[69] Bryant JA, Stewart FJ, Eppley JM, DeLong EF (2012) Microbial community phylogenetic and trait diversity declines with depth in a marine oxygen minimum zone. Ecology 93:1659–1673. 10.1890/11-1204.1 22919912 10.1890/11-1204.1

[70] O’Dwyer JP, Kembel SW, Green JL (2012) Phylogenetic diversity theory sheds light on the structure of microbial communities. PLoS Comput Biol 8:e1002832. 10.1371/journal.pcbi.1002832 23284280 10.1371/journal.pcbi.1002832 PMC3527210

[71] Paver SF, Muratore D, Newton RJ, Coleman ML (2018) Reevaluating the salty divide: phylogenetic specificity of transitions between marine and freshwater systems. mSystems 3:10.1128/msystems.00232 − 18. 10.1128/msystems.00232-18 10.1128/mSystems.00232-18 PMC6234284 30443603

[72] Tamames J, Abellán JJ, Pignatelli M et al (2010) Environmental distribution of prokaryotic taxa. BMC Microbiol 10:85. 10.1186/1471-2180-10-85 20307274 10.1186/1471-2180-10-85 PMC2850351

[73] Kelly WR, Panno SV, Hackley KC et al (2009) Bacteria contamination of groundwater in a mixed land-use karst region. Water Qual Expo Health 1:69–78. 10.1007/s12403-009-0006-7

[74] Davis MC, Messina MA, Nicolosi G et al (2020) Surface runoff alters cave microbial community structure and function. PLoS One 15:e0232742. 10.1371/journal.pone.0232742 32374788 10.1371/journal.pone.0232742 PMC7202643

[75] Farkas A, Coman C, Szekeres E et al (2022) Molecular typing reveals environmental dispersion of antibiotic-resistant enterococci under anthropogenic pressure. Antibiotics Basel 11:1213. 10.3390/antibiotics11091213 36139992 10.3390/antibiotics11091213 PMC9494986

[76] Felföldi T, Ramganesh S, Somogyi B et al (2016) Winter planktonic microbial communities in highland aquatic habitats. Geomicrobiol J 33:494–504. 10.1080/01490451.2015.1059523

[77] Worthington SRH, Smart CC (2017) Transient bacterial contamination of the dual-porosity aquifer at Walkerton, Ontario, Canada. Hydrogeol J 25:1003–1016. 10.1007/s10040-016-1514-8

[78] Buckerfield SJ, Waldron S, Quilliam RS et al (2019) How can we improve understanding of faecal indicator dynamics in karst systems under changing climatic, population, and land use stressors? – Research opportunities in SW China. Sci Total Environ 646:438–447. 10.1016/j.scitotenv.2018.07.292 30056232 10.1016/j.scitotenv.2018.07.292

[79] Vucinic L, O’Connell D, Teixeira R et al (2022) Flow cytometry and fecal indicator bacteria analyses for fingerprinting microbial pollution in Karst aquifer systems. Water Resour Res 58:e2021WR029840. 10.1029/2021WR029840 35859924 10.1029/2021WR029840 PMC9285701

[80] Daszak P, Cunningham AA, Hyatt AD (2001) Anthropogenic environmental change and the emergence of infectious diseases in wildlife. Acta Trop 78:103–116. 10.1016/S0001-706X(00)00179-0 11230820 10.1016/s0001-706x(00)00179-0

[81] O’Gorman EJ, Fitch JE, Crowe TP (2012) Multiple anthropogenic stressors and the structural properties of food webs. Ecology 93:441–448. 10.1890/11-0982.1 22624198 10.1890/11-0982.1

[82] Eckert EM, Quero GM, Di Cesare A et al (2019) Antibiotic disturbance affects aquatic microbial community composition and food web interactions but not community resilience. Molecular Ecology 28:1170–1182. 10.1111/mec.15033 30697889 10.1111/mec.15033

[83] Ghaly TM, Focardi A, Elbourne LDH et al (2023) Stratified microbial communities in Australia’s only anchialine cave are taxonomically novel and drive chemotrophic energy production via coupled nitrogen-sulphur cycling. Microbiome 11:190. 10.1186/s40168-023-01633-8 37626351 10.1186/s40168-023-01633-8 PMC10463829

[84] Kovacs SE, van Hengstum PJ, Reinhardt EG et al (2013) Late Holocene sedimentation and hydrologic development in a shallow coastal sinkhole on Great Abaco Island, The Bahamas. Quaternary International 317:118–132. 10.1016/j.quaint.2013.09.032

[85] Tamalavage AE, van Hengstum PJ, Louchouarn P et al (2018) Organic matter sources and lateral sedimentation in a Bahamian karst basin (sinkhole) over the late Holocene: influence of local vegetation and climate. Palaeogeography, Palaeoclimatology, Palaeoecology 506:70–83. 10.1016/j.palaeo.2018.06.014

[86] Gonzalez BC, Iliffe TM, Macalady JL et al (2011) Microbial hotspots in anchialine blue holes: initial discoveries from the Bahamas. Hydrobiologia 677:149–156. 10.1007/s10750-011-0932-9

[87] Havird JC, Brannock PM, Yoshioka RM et al (2022) Grazing by an endemic atyid shrimp controls microbial communities in the Hawaiian anchialine ecosystem. Limnology and Oceanography 67:2012–2027. 10.1002/lno.12184

[88] Mejía-Ortíz LM, Chávez-Solís EM, Brankovits D (2022) Editorial: The effects of environmental change on anchialine ecosystems. Front Mar Sci 9. 10.3389/fmars.2022.1029027

[89] Izquierdo T (2014) Conceptual hydrogeological model and aquifer system classification of a small volcanic island (La Gomera; Canary Islands). CATENA 114:119–128. 10.1016/j.catena.2013.11.006

[90] García Jiménez MDP, Carrasco Acosta M, Hettmann S (2020) Halobacterium identification in saltworks of Gran Canaria (Canary Islands, Spain). J fisheries Sci 2:9–16. 10.30564/jfsr.v2i1.1464

[91] European Commission (2000) Directive 2000/60/EC of the European Parliament and of the Council of 23 October 2000 establishing a framework for. Community action in the field of water policy

[92] Mammola S, Cardoso P, Culver DC et al (2019) Scientists’ warning on the conservation of subterranean ecosystems. BioScience 69:641–650. 10.1093/biosci/biz064

[93] Di Lorenzo T, Lunghi E, Aanei C et al (2024) EU needs groundwater ecosystems guidelines. Science 386:1103–1103. 10.1126/science.ads8140 39636973 10.1126/science.ads8140

[94] Oromí P, Arechavaleta M, García R et al (2021) Diversidad faunística en el medio subterráneo volcánico, con especial énfasis en las Islas Canarias. Boletín de la Sociedad Española de Espeleología y Ciencias del Karst

