# Supplementary material for: Multilayered Human Activities Shape the Microbial Communities of Groundwater-Dependent Ecosystems on an Arid Oceanic Island
Source: Microb Ecol. 2026 Jun 8;89:157. doi: 10.1007/s00248-026-02797-0 (PMC13461696; doi:10.1007/s00248-026-02797-0)
Supplement: Supplementary file 1 [file 248_2026_2797_MOESM2_ESM.docx]

**Supplementary Material for**

**Multilayered human activities shape the microbial communities of groundwater-dependent ecosystems on an arid oceanic island**

Francesco Di Nezio^1^*, Andrea Di Cesare^1,2^, Marta García-Cobo^1,3^, David Brankovits^1^, Raffaella Sabatino^1,2^, Giulia Borgomaneiro^1^, Zacarías Fresno-López^1^, Márcia Neunschwander Kurtz^1^, Sarah Boulamail^4^, Francesco Cozzoli^4^, Lara Fumarola^5^, Brett C. Gonzalez^6^, Alvaro Roldán^7^, Cristina Camacho^8^, Alvaro García-Herrera^9^, Leopoldo Moro^10^, Concepcion Valdivia^10^, Elena Mateo^11^, Guillermo García-Gómez^12^, Diego Fontaneto^1,2^, Gianluca Corno^1,2^, Ester M. Eckert^1,2^**, Alejandro Martínez^1^**

^1^ National Research Council of Italy (CNR) – Water Research Institute (IRSA), Verbania, Italy

^2^ National Biodiversity Future Center (NBFC), Palermo, Italy.

^3^ Universidad Complutense de Madrid, Department of Biodiversity, Ecology and Evolution, Madrid, Spain

^4^ National Research Council of Italy (CNR) – Research Institute on Terrestrial Ecosystems (IRET), Lecce, Italy

^5^ University of Salento, Department of Biological and Environmental Sciences and Technologies (Di.S.Te.B.A), Lecce, Italy

^6^ The University of Western Australia, School of Biological Sciences, Perth, Australia

^7^ Technical and Scientific diving freelance, Las Breñas, Yaiza, Spain

^8^ Freelance, Puerto del Carmen, Tías, Las Palmas, Spain

^9^ Asesores Técnicos de Medio Ambiente ATECMA SL, Collado Villalba, Spain

^10^ Servicio de Biodiversidad, Gobierno de Canarias, Santa Cruz de Tenerife, Spain

^11^ Lanzarote and Chinijo Islands UNESCO Global Geopark, Mozaga, Spain

^12^ Rey Juan Carlos University, Global Change Research Institute (IICG), Madrid, Spain

**Supplementary Text 1**

**Site Descriptions**

*Cave Systems*

Caves were represented by eight samples collected from different sections of La Corona lava tube: Cueva de los Lagos (Figure S1A), Jameos del Agua (Figure S1B), and several sections of Túnel de la Atlántida (Figure S1D, entrance pool, La Sima, Lago Escondido, Montaña de Arena). All provide access to the island’s basal aquifer, which dates to the island’s formation (15 million years ago), though the lava tube itself formed only 20,000 years ago. These sites span a gradient of human influence, from low visitation at Cueva de los Lagos (50–100 visitors annually) to highly touristic locations in Los Jameos del Agua, inaugurated in 1977 and receiving >800,000 visitors per year [1]. Samples from Túnel de la Atlántida were collected along a gradient of increasing distance from the cave entrance at the touristic center, extending to La Sima (~150 m), Lago Escondido (~250 m), and Montaña de Arena (~750 m). Los Jameos del Agua receives limited sunlight whereas, except for its entrance, all cave locations in the Túnel de la Atlántida are in complete darkness [2].

*Anchialine Pools*

Anchialine pools are tidally influenced inland pools flooded by saline groundwater from the island’s basal aquifer but exposed to light and receiving a strong terrestrial and marine influence through the entrance of particulate organic matter and sea spray. We sampled those at Charcos de Luis (Figure S1E), Charcos Grande and Pequeño de Montaña Bermeja (Figure S1F), Charco de Suso, Charco de Zac, and Charco de Punta Prieta. While most of these pools remain largely undisturbed, Montaña Bermeja is popular for recreational use, and Charco de Zac serves local fishermen as a holding area for bait and fish.

*Saltworks*

Saltworks were represented by the Salinas de los Agujeros, the only active saltworks still supplied by saline groundwater from the island’s basal aquifer. We collected samples from two hypersaline saltpans (Figure S1K), exposed to sunlight and receiving strong terrestrial input; as well as an abandoned pumping well that showed evidence of fuel contamination (Figure S1J).

*Groundwater Wells, Galleries, and Spring-fed Ponds*

Groundwater wells, galleries, and spring-fed ponds were sampled at Tabayesco, Famara, and Haría. These sites access the fresh- or slightly brackish waters of perched aquifers along the mountain ranges of the island, whose formation dates to earlier geological stages, when mountain ridges intercepted moist Tradewinds and hosted dense laurel forests. Today, these wells and mines continue to supply water for domestic and agricultural use, drawing from captured springs, open tanks, and active pumping systems. Wells in Tabayesco and Haría are still regularly used by local farmers and shepherds. The bacterial communities likely reflect a mixture of environmental taxa from both the aquifer and surrounding habitats, as well as anthropogenic taxa introduced through direct human interaction and indirect influences on the aquifer. Local sites in Famara are likely affected by marine spray (Figure S1H), while those in Tabayesco are more influenced by inland conditions and recurrent human and livestock activity. The well in Haría is situated within an urban setting, potentially subject to additional urban-associated impacts (Figure S1I). Although wastewater from Haría is treated at the Haría–Arrieta wastewater treatment plant (WWTP), many houses built before the early 20th century still use independent cesspits instead of connection to the municipal sewer network. Although these habitats differ in environmental context and human impact, all are classified as wells. The only exception was the freshwater spring-fed pond in Tabayesco, which we categorized separately, due to its high exposure to sunlight, rainfall, and external disturbances (Figure S1C).

*Coastal Environments and Bays*

Coastal environments and enclosed bays influenced by submarine groundwater discharge (SGD) were sampled across three distinct areas of the island, each characterized by a different level of anthropogenic impact. Samples at Caletón Blanco were collected near the small town of Órzola (352 inhabitants, as of 2021), which first appears in maps from the 17th century [3] as a small fishermen settlement built around a saltwork complex, nowadays inactive. The village is far from any WWTP and, therefore, the wastewater is likely to be discharged directly into the ocean or collected in fecal wells. At Charco de San Ginés (Figure S1G), located in the island’s capital Arrecife, samples were taken in an enclosed bay within the city. Although the bay is clearly enriched with organic matter from the fishermen’s activities, there is no indication of wastewater discharge in the area, as the city is equipped with functioning WWTP. In contrast, samples at Punta del Usaje were collected within the legally protected area of Malpaís de la Corona, a remote zone with no nearby human settlements and strict regulations prohibiting wastewater discharge.

**Supplementary Text 2**

**Additional sequencing details and protocols**

PCR reactions (25 µL) included microbial genomic DNA, forward and reverse primers, and KAPA HiFi HotStart ReadyMix. Thermal cycling conditions consisted of an initial denaturation at 95 °C for 3 min, followed by denaturation, annealing, and extension steps, and a final extension at 72 °C for 5 min.

PCR products were purified using AMPure XP beads prior to index PCR amplification with the Nextera XT Index Kit. Index PCR reactions (50 µL) included purified amplicons, Nextera XT index primers, KAPA HiFi HotStart ReadyMix, and PCR-grade water. Thermal cycling consisted of an initial denaturation at 95 °C for 3 min, followed by amplification cycles and a final extension at 72 °C for 5 min.

Indexed libraries were purified with AMPure XP beads, quantified fluorometrically, and pooled prior to sequencing.

**Sequence quality filtering**

Sequences with ambiguous bases were discarded, and the maximum expected errors were set to 2 for forward reads and 4 for reverse reads. Reads with a quality score below 2 were removed, and PhiX contamination was filtered out by setting rm.phix = TRUE in DADA2’s filterAndTrim() function, which filters sequences matching the PhiX174 genome. These parameters were chosen based on inspection of quality score profiles using the plotQualityProfile() function. Reads were then merged to generate unique Amplicon Sequence Variants (ASVs). To minimize the influence of sequencing artefacts and extremely rare variants, the ASV table was further filtered by removing ASVs occurring in fewer than two samples or represented by fewer than three reads across the dataset. All downstream analyses were performed on this filtered ASV table. Consequently, ASV richness values reported in this study represent conservative estimates of supported ASV richness rather than raw unfiltered richness.

**Supplementary Text 3**

Structured query framework used to interrogate Copilot AI for the assignment of putative taxon origin, reported here in bash-style pseudocode for transparency and reproducibility. The prompt summarizes the logic described in the Materials and Methods, including the database categories considered, the aggregation of matches across environmental, wastewater, and human-associated records, and the rule-based assignment of taxa based on proportion and minimum-match thresholds.

*#!/usr/bin/env bash*

*# CNR-IRSA Version - Integrated Origin Assignment (Stochastic Ratio 2:1:1)*

*# -----------------------------------------------------------------------*

*# Usage: ./script.sh species_list.txt metagenomes_dataset.txt*

*# -----------------------------------------------------------------------*

*set -euo pipefail*

*# --- CONFIGURATION ---*

*STOP_THRESHOLD=400*

*THRESHOLD_PROP="0.90"*

*OUT_ROOT="results_origin"*

*SUMMARY_DIR="${OUT_ROOT}/summary"*

*TEMP_DIR="${OUT_ROOT}/temp_split"*

*mkdir -p "$SUMMARY_DIR" "$TEMP_DIR"*

*# --- INPUT CHECK ---*

*if [[ $# -lt 2 ]]; then*

*echo "Error: Missing arguments."*

*echo "Usage: $0 <species_list.txt> <metagenomes_dataset.txt>"*

*exit 1*

*fi*

*SPECIES_FILE="$1"*

*DATASET_FILE="$2"*

*MG_FOLDER="$3"*

*# --- DATASET PREPARATION --- Splitting the 4000 metagenomes into group-specific temp files*

*prepare_datasets() {*

*echo "Organizing metagenome dataset by category..."*

*grep "ENVIRONMENTAL" "$DATASET_FILE" | cut -d',' -f1 > "${TEMP_DIR}/list_env.txt"*

*grep "WASTEWATER" "$DATASET_FILE" | cut -d',' -f1 > "${TEMP_DIR}/list_wwt.txt"*

*grep "HUMAN" "$DATASET_FILE" | cut -d',' -f1 > "${TEMP_DIR}/list_hum.txt"*

*ENV_COUNT=$(wc -l < "${TEMP_DIR}/list_env.txt")*

*WWT_COUNT=$(wc -l < "${TEMP_DIR}/list_wwt.txt")*

*HUM_COUNT=$(wc -l < "${TEMP_DIR}/list_hum.txt")*

*echo "Dataset loaded: $ENV_COUNT Env, $WWT_COUNT WWT, $HUM_COUNT Hum."*

*}*

*# --- SEARCH ENGINE (GREP) --- taxon string inside the specific metagenome file*

*check_presence() {*

*local taxon="$1"*

*local mg_id="$2"*

*local file_path="${MG_FOLDER}/${mg_id}.txt"*

*if [[ ! -f "$file_path" ]]; then*

*echo 0*

*return*

*fi*

*# Grep search: -q (quiet) returns 0 if found, 1 if not. We invert it to return 1 for "found" and 0 for "not found" for our counter.*

*if grep -q "$taxon" "$file_path"; then*

*echo 1*

*else*

*echo 0*

*fi*

*}*

*# --- CORE ANALYSIS ENGINE ---*

*process_taxon() {*

*local taxon="$1"*

*# Shuffle indices for stochastic sampling*

*shuf "${TEMP_DIR}/list_env.txt" > "${TEMP_DIR}/shuf_env.txt"*

*shuf "${TEMP_DIR}/list_wwt.txt" > "${TEMP_DIR}/shuf_wwt.txt"*

*shuf "${TEMP_DIR}/list_hum.txt" > "${TEMP_DIR}/shuf_hum.txt"*

*local h_env=0 h_wwt=0 h_hum=0 h_total=0*

*local n_env=0 n_wwt=0 n_hum=0*

*local p_env=1 p_wwt=1 p_hum=1*

*local method="DATASET_EXHAUSTED"*

*# Iterative 2:1:1 Sampling Loop and Exit if all available samples are checked*

*while [[ $h_total -lt $STOP_THRESHOLD ]]; do*

*if [[ $p_env -gt $ENV_COUNT && $p_wwt -gt $WWT_COUNT && $p_hum -gt $HUM_COUNT ]]; then break; fi*

*for i in {1..2}; do*

*if [[ $p_env -le $ENV_COUNT && $h_total -lt $STOP_THRESHOLD ]]; then*

*id=$(sed -n "${p_env}p" "${TEMP_DIR}/shuf_env.txt")*

*[[ $(check_presence "$taxon" "$id") -eq 1 ]] && ((h_env++)) && ((h_total++))*

*((n_env++)); ((p_env++))*

*fi*

*done*

*if [[ $p_wwt -le $WWT_COUNT && $h_total -lt $STOP_THRESHOLD ]]; then*

*id=$(sed -n "${p_wwt}p" "${TEMP_DIR}/shuf_wwt.txt")*

*[[ $(check_presence "$taxon" "$id") -eq 1 ]] && ((h_wwt++)) && ((h_total++))*

*((n_wwt++)); ((p_wwt++))*

*fi*

*if [[ $p_hum -le $HUM_COUNT && $h_total -lt $STOP_THRESHOLD ]]; then*

*id=$(sed -n "${p_hum}p" "${TEMP_DIR}/shuf_hum.txt")*

*[[ $(check_presence "$taxon" "$id") -eq 1 ]] && ((h_hum++)) && ((h_total++))*

*((n_hum++)); ((p_hum++))*

*fi*

*[[ $h_total -eq $STOP_THRESHOLD ]] && method="TARGET_REACHED"*

*done*

*# Final Statistics*

*calc_p() { awk "BEGIN { if ($2==0) print 0; else printf \"%.4f\", ($1/$2) }"; }*

*local pr_env=$(calc_p "$h_env" "$h_total")*

*local pr_wwt=$(calc_p "$h_wwt" "$h_total")*

*local pr_hum=$(calc_p "$h_hum" "$h_total")*

*local origin="INDETERMINATE"*

*if (( $(echo "$pr_env >= $THRESHOLD_PROP" | bc -l) )); then origin="ENVIRONMENTAL";*

*elif (( $(echo "$pr_wwt >= $THRESHOLD_PROP" | bc -l) )); then origin="WASTEWATER";*

*elif (( $(echo "$pr_hum >= $THRESHOLD_PROP" | bc -l) )); then origin="HUMAN"; fi*

*printf "%s,%d,%d,%d,%d,%d,%d,%d,%s,%s\n" \*

*"$taxon" "$h_env" "$h_wwt" "$h_hum" "$h_total" "$n_env" "$n_wwt" "$n_hum" "$method" "$origin"*

*}*

*# --- MAIN ---*

*prepare_datasets*

*REPORT="${SUMMARY_DIR}/final_origin_assignment.csv"*

*echo "taxon,hits_env,hits_wwt,hits_hum,total_hits,n_env,n_wwt,n_hum,method,assigned_origin" > "$REPORT"*

*echo "Starting analysis for species in $SPECIES_FILE..."*

*while IFS= read -r taxon || [[ -n "$taxon" ]]; do*

*[[ -z "$taxon" ]] && continue*

*echo "Processing: $taxon"*

*process_taxon "$taxon" >> "$REPORT"*

*done < "$SPECIES_FILE"*

*echo "-------------------------------------------------------"*

*echo "Analysis Complete. Report: $REPORT"*

**Supplementary Text 4**

**Microbial dynamics in cave ecosystems**

To investigate habitat-specific dynamics in greater depth, we conducted a focused analysis of cave microbiomes, treating these semi-enclosed environments as ecological enclaves. Caves displayed distinct patterns in microbial richness, composition, and functional group structure, influenced primarily by light availability and proximity to the sea (Figure S2). We followed the same methods as for the entire dataset, but including the following covariates:

A likelihood ratio test (Table S12) revealed that both light regime (p < 0.001) and distance from the sea (p = 0.005) significantly predicted bacterial richness. Pairwise comparisons (Table S13) showed that twilight zones - areas with partial light exposure - hosted significantly more diverse communities than either fully dark (p = 0.0002) or fully sunlit sites (p < 0.0001). These intermediate-light areas appear to act as microbial diversity hotspots, likely benefiting from stable humidity, moderate temperatures, and low disturbance. Richness also declined with increasing distance from the sea, suggesting that marine connectivity may contribute to cave microbiome structure, potentially through saltwater intrusion or coastal runoff.

Despite these richness gradients, community composition remained relatively stable along cave transects. PERMANOVA revealed that none of the measured environmental variables significantly influenced total β-diversity (all p > 0.1; Table S14), indicating spatial consistency in microbial assemblages. However, partitioned β-diversity analyses showed a marginal effect of light on nestedness (p = 0.064), suggesting that light availability may influence selective loss or retention of taxa, rather than community turnover per se.

Importantly, caves harbored the highest load of pathogenic and human-associated bacteria across all habitats. Several clinically relevant genera were frequently detected in multiple cave subsites, including both entrances and deeper twilight areas. Pathogen prevalence was markedly higher in caves than in open aquatic systems, including coastal and saltworks habitats, which consistently showed low or negligible pathogen signals. Hierarchical clustering based on pathogenic and anthropogenic taxa (Figure S4) further emphasized this pattern, consistently grouping cave samples apart from cleaner marine environments.

**Supplementary Text 5**

**Phylogenetic diversity calculation**

To estimate phylogenetic diversity among bacterial assemblages, we reconstructed a phylogenetic tree from the aligned 16S ASVs sequences using the MAFFT version 7 [12], allowing the software to choose the optimal alignment optimal strategy based on our dataset. We then compute pairwise genetic distances and a neighbor-joining tree using the functions dist.dna and njs from the package *ape* version 5.0 [13]. We set the option pairwise deletion as true to handle missing data. Faith’s Phylogenetic Diversity was then computed for each sample using the function ‘alpha’ included in BAT version 2.9.5 [8]. To assess patterns of phylogenetic composition among samples, we calculate pairwise β-diversity metrics using the function ‘beta’ in *BAT*. To evaluate environmental correlations of phylogenetic richness, we implemented generalized linear models with a Gamma distribution and log link, using the same model structure as for the taxonomic diversity (see Methods). Model assumptions were checked using the ‘check_model’ function in the package *performance* version 0.11 [5]. Pairwise post hoc contrasts between habitat types were computed with Tukey adjustment using the emmeans package. Differences in total, nestedness, and turnover ASVs composition components across habitat types were tested using permutational multivariate analysis of variance (PERMANOVA) via using the function adonis2 in *vegan* [9] using the same model as above. We set 9999 permutations and “by = terms”. We visualize within- and between-habitat differences in β-diversity using density plots.

**Phylogenetic diversity results**

Phylogenetic diversity (PD) also varied significantly with habitat type (χ² = 14.7, p = 0.012; Table S15) with all the other covariates having no significant effect (all p > 0.2). Post hoc pairwise comparisons showed that caves harbored higher PD than wells, reflecting both deeper evolutionary lineages and greater overall richness. All other comparisons were non-significant (Table S16). Phylogenetic β-diversity further supported the structuring role of habitat. PERMANOVA revealed a significant effect of habitat type on total phylogenetic β-diversity (p = 0.011; Table S17), indicating that microbial lineages vary substantially among environments.

Unlike taxonomic β-diversity, which was driven by turnover, phylogenetic differences were more influenced by nestedness, although marginally non-significant (p = 0.063; Figure S3). This suggests that microbial communities across habitats remain evolutionarily connected, but selective filtering leads to the persistence of only specific clades under restrictive conditions. Wells retained only shallow subsets of lineages, while caves preserved phylogenetically diverse assemblages.

**Table S1** Overview of groundwater-dependent coastal habitats in Lanzarote, summarizing their associated aquifer types, representative sites, main anthropogenic pressures, key ecosystem services, and proposed conservation strategies. For detailed habitat descriptions and additional examples, see Figure 1 and Table 1. (SGD = submarine groundwater discharge; WWTPs = wastewater treatment plants)

| **Habitat** | **Anthropogenic pressures** | **Ecosystem Services** | **Proposed conservation actions** | **References** |
| --- | --- | --- | --- | --- |
| ***Perched freshwater aquifers (salinity: 0.8 - 3.6 ‰)*** | | | | |
| Mountain and interior wells and spring-fed ponds | Canalization of springs; construction of wells and galleries; groundwater extraction; organic enrichment; pathogens from livestock and human use | Drinking water; wetlands  groundwater dependent vegetation | Monitoring of groundwater extraction; protection of recharge areas; restoration of natural flow paths; sanitation management near springs; regular microbiological and physicochemical monitoring; citizen science monitoring initiatives for private wells; collaboration with Lanzarote’s island council | [14, 15] |
| ***Basal saline aquifer (salinity: 36.0 - 95.8 ‰)*** | | | | |
| Lowland wells and impermeable pools | Construction of wells, saltworks, and saltpans; water and fauna extraction; artificial wetlands; human presence, pollution, pathogens | Salt production; wetlands (bird breeding) | Regulate groundwater extraction; maintain traditional saltworks sustainably; control pollutants; promote eco-tourism; promote laws for periodical groundwater evaluation; citizen science monitoring initiatives for private wells | [16, 17] |
| Anchialine caves | Tourist visitation and caving; human pathogens; coin disposal; infrastructure-derived pollution | Tourism; Scientific research; Science communication; Cultural activities | Access control and visitor quotas; regular microbiological and physicochemical monitoring; environmental education; inclusion in protected area management | [18, 19] |
| Anchialine pools | Pollution from visitors, surface runoff, or nearby infrastructure | Temporary fish reservoirs; wetlands; Groundwater dependent vegetation; recreational use | Habitat fencing or visitor control; runoff mitigation; periodic water quality assessment; local stewardship initiatives | [20] |
| Submarine groundwater discharge in coastal bays | Nutrient enrichment from SGD; eutrophication risk; contamination (nitrates, pathogens); WWTPs; altered nearshore food webs; degradation of seagrass and rocky reef habitats | Fisheries; coastal productivity; recreation; tourism | Integrated coastal groundwater management; nutrient input reduction; monitoring of SGD plumes; protection of seagrass and reef habitats | [21, 22] |

**Table S2** Counts and percentages of ASVs and reads assigned to Archaea, mitochondria, chloroplasts, and sequences unassigned at the phylum level, relative to the pre-filtered dataset. Percentages are calculated relative to the total number of ASVs and reads in the pre-filtered dataset

| **Category** | **ASVs** | **Reads** | **ASVs (%)** | **Reads (%)** |
| --- | --- | --- | --- | --- |
| Archaea | 359 | 42228 | 2.16 | 3.75 |
| Mitochondria | 21 | 198 | 0.13 | 0.02 |
| Chloroplast | 176 | 27646 | 1.06 | 2.46 |
| Unassigned at phylum level | 369 | 7653 | 2.22 | 0.68 |

**Table S5.** Pairwise Tukey-adjusted comparisons of habitat types for total microbial richness, measured as number of ASVs per sample, based on estimated marginal means (EMMs) from the Negative Binomial model. Contrasts were computed using the emmeans package on the log scale.

| **Contrast** | **Estimate** | **SE** | **Df** | **z ratio** | **p value** |
| --- | --- | --- | --- | --- | --- |
| cave - pond | 0.643 | 0.646 | Inf | 0.995 | 0.920 |
| cave - pool | 0.816 | 0.248 | Inf | 3.291 | 0.013 |
| cave - salt | 0.891 | 0.514 | Inf | 1.735 | 0.509 |
| cave - sea | 0.300 | 0.303 | Inf | 0.988 | 0.922 |
| cave - well | 1.594 | 0.304 | Inf | 5.249 | <.0001 |
| pond - pool | 0.173 | 0.662 | Inf | 0.261 | 1.000 |
| pond - salt | 0.249 | 0.808 | Inf | 0.308 | 1.000 |
| pond - sea | -0.343 | 0.695 | Inf | -0.493 | 0.996 |
| pond - well | 0.951 | 0.588 | Inf | 1.618 | 0.587 |
| pool - salt | 0.076 | 0.521 | Inf | 0.145 | 1.000 |
| pool - sea | -0.516 | 0.313 | Inf | -1.649 | 0.566 |
| pool - well | 0.778 | 0.327 | Inf | 2.379 | 0.164 |
| salt - sea | -0.592 | 0.429 | Inf | -1.378 | 0.741 |
| salt - well | 0.703 | 0.531 | Inf | 1.324 | 0.772 |
| sea - well | 1.294 | 0.368 | Inf | 3.517 | 0.006 |

**Table S6.** Linear model results for Shannon diversity across habitats. Model significance was assessed using Type II ANOVA.

| **Estimate** | **Std. Error** | **Sum Sq** | **Df** | **F value** | **Pr(>F)** |
| --- | --- | --- | --- | --- | --- |
| Type | NA | NA | 13.738 | 5 | 3.656 |
| reads | -1.78E-05 | 1.91E-05 | 0.656 | 1 | 0.872 |
| Distance from sea | 4.87E-04 | 4.51E-04 | 0.879 | 1 | 1.169 |
| Nr. of buildings | 0.048 | 0.141 | 0.088 | 1 | 0.117 |
| Residuals | NA | NA | 14.28 | 19 |  |

**Table S7.** Pairwise comparisons of Shannon diversity among habitats (Tukey-adjusted p-values) based on the linear model. Estimates represent differences between habitat pairs.

| **contrast** | **estimate** | **SE** | **df** | **t.ratio** | **p.value** |
| --- | --- | --- | --- | --- | --- |
| sea - cave | -0.643 | 0.600 | 19 | -1.072 | 0.887 |
| sea - pool | 0.478 | 0.634 | 19 | 0.754 | 0.972 |
| sea - salt | 0.025 | 0.829 | 19 | 0.030 | 1.000 |
| sea - well | 1.673 | 0.686 | 19 | 2.439 | 0.192 |
| sea - pond | 1.466 | 1.390 | 19 | 1.057 | 0.892 |
| cave - pool | 1.121 | 0.469 | 19 | 2.388 | 0.209 |
| cave - salt | 0.668 | 1.030 | 19 | 0.650 | 0.985 |
| cave - well | 2.317 | 0.571 | 19 | 4.060 | **0.008** |
| cave - pond | 2.110 | 1.230 | 19 | 1.718 | 0.537 |
| pool - salt | -0.453 | 1.060 | 19 | -0.429 | 0.998 |
| pool - well | 1.196 | 0.614 | 19 | 1.948 | 0.405 |
| pool - pond | 0.989 | 1.260 | 19 | 0.787 | 0.966 |
| salt - well | 1.649 | 1.020 | 19 | 1.610 | 0.602 |
| salt - pond | 1.442 | 1.650 | 19 | 0.875 | 0.948 |
| well - pond | -0.207 | 1.130 | 19 | -0.184 | 1.000 |

**Table S8.** Pairwise comparisons of habitat types for bacterial functional groups reads abundance, obtained from estimated marginal means (EMMs) computed on the betabinomial GLM. Tukey-adjusted contrasts were calculated using the emmeans framework on the log-odds scale.

| **Potentially pathogenic bacteria** | | | | | | **Human-related bacteria** | | | | | | **Environmental bacteria** | | | | | |
| --- | --- | --- | --- | --- | --- | --- | --- | --- | --- | --- | --- | --- | --- | --- | --- | --- | --- |
| **Contrast** | **Estimate** | **SE** | **Df** | **z ratio** | **p value** | **Contrast** | **Estimate** | **SE** | **Df** | **z ratio** | **p value** | **Contrast** | **Estimate** | **SE** | **Df** | **z ratio** | **p value** |
| cave - pond | -2.599 | 0.406 | Inf | -6.406 | **<.0001** | cave - pond | -1.372 | 0.622 | Inf | -2.206 | 0.235 | cave - pond | -0.098 | 0.305 | Inf | -0.320 | 0.999 |
| cave - pool | 1.167 | 1.070 | Inf | 1.086 | 0.887 | cave - pool | -0.968 | 1.680 | Inf | -0.575 | 0.993 | cave - pool | -0.422 | 0.651 | Inf | -0.648 | 0.987 |
| cave - salt | -0.051 | 0.463 | Inf | -0.111 | 1.000 | cave - salt | -0.343 | 0.611 | Inf | -0.562 | 0.993 | cave - salt | 0.300 | 0.319 | Inf | 0.939 | 0.936 |
| cave - sea | 0.239 | 0.574 | Inf | 0.416 | 0.998 | cave - sea | 0.270 | 0.814 | Inf | 0.331 | 1.000 | cave - sea | -0.322 | 0.377 | Inf | -0.856 | 0.957 |
| cave - well | -0.146 | 0.555 | Inf | -0.262 | 1.000 | cave - well | -1.255 | 0.818 | Inf | -1.533 | 0.643 | cave - well | 1.225 | 0.446 | Inf | 2.747 | 0.066 |
| pond - pool | 3.766 | 1.020 | Inf | 3.691 | **0.003** | pond - pool | 0.404 | 1.520 | Inf | 0.266 | 1.000 | pond - pool | -0.324 | 0.631 | Inf | -0.514 | 0.996 |
| pond - salt | 2.548 | 0.357 | Inf | 7.128 | **<.0001** | pond - salt | 1.028 | 0.524 | Inf | 1.962 | 0.365 | pond - salt | 0.398 | 0.246 | Inf | 1.615 | 0.589 |
| pond - sea | 2.838 | 0.609 | Inf | 4.657 | **<.0001** | pond - sea | 1.642 | 0.932 | Inf | 1.761 | 0.491 | pond - sea | -0.225 | 0.461 | Inf | -0.488 | 0.997 |
| pond - well | 2.453 | 0.399 | Inf | 6.146 | **<.0001** | pond - well | 0.117 | 0.590 | Inf | 0.199 | 1.000 | pond - well | 1.323 | 0.353 | Inf | 3.745 | 0.003 |
| pool - salt | -1.218 | 1.060 | Inf | -1.152 | 0.859 | pool - salt | 0.625 | 1.600 | Inf | 0.391 | 0.999 | pool - salt | 0.722 | 0.652 | Inf | 1.108 | 0.878 |
| pool - sea | -0.928 | 1.160 | Inf | -0.798 | 0.968 | pool - sea | 1.238 | 1.850 | Inf | 0.671 | 0.985 | pool - sea | 0.100 | 0.708 | Inf | 0.141 | 1.000 |
| pool - well | -1.312 | 0.946 | Inf | -1.387 | 0.735 | pool - well | -0.287 | 1.340 | Inf | -0.214 | 1.000 | pool - well | 1.647 | 0.611 | Inf | 2.697 | 0.076 |
| salt - sea | 0.290 | 0.639 | Inf | 0.454 | 0.998 | salt - sea | 0.613 | 0.903 | Inf | 0.679 | 0.984 | salt - sea | -0.622 | 0.470 | Inf | -1.324 | 0.772 |
| salt - well | -0.094 | 0.507 | Inf | -0.186 | 1.000 | salt - well | -0.911 | 0.699 | Inf | -1.304 | 0.783 | salt - well | 0.925 | 0.378 | Inf | 2.450 | 0.139 |
| sea - well | -0.384 | 0.720 | Inf | -0.534 | 0.995 | sea - well | -1.524 | 1.090 | Inf | -1.395 | 0.730 | sea - well | 1.548 | 0.570 | Inf | 2.714 | 0.072 |

**Table S9** Inference patterns of proportional richness of potentially pathogenic, human-related, and environmental bacteria, modelled using generalized linear models under a logit binomial link. The models were fitted using richness as the response and habitat type, sequencing depth (reads), distance from the sea, and local building density (log(buildings + 1)) as predictors. (Std = standard, Df = degrees of freedom)

| **Proportionl richness of potentially pathogenic bacteria** | | | | | | |
| --- | --- | --- | --- | --- | --- | --- |
|  | **Estimate** | **Std. Error** | **z value** | **LR Chisq** | **Df** | **Pr(>Chisq)** |
| Type | NA | NA | NA | 59.890 | 5 | **< 0.0001** |
| Reads | 1.05E-05 | 5.16E-05 | 2.050 | 4.239 | 1 | **0.039** |
| Nr. of buildings | 0.051 | 0.048 | 1.134 | 1.273 | 1 | 0.259 |
| **Proportional richness of human-related bacteria** | | | | | | |
|  | **Estimate** | **Std. Error** | **z value** | **LR Chisq** | **Df** | **Pr(>Chisq)** |
| Type | NA | NA | NA | 33.924 | 5 | **< 0.0001** |
| Reads | -2.65E-07 | 5.13E-06 | -0.052 | 0.003 | 1 | 0.964 |
| Nr. of buildings | 0.074 | 0.042 | 1.773 | 3.149 | 1 | 0.075 |
| **Proportional richness of environmental bacteria** | | | | | | |
|  | **Estimate** | **Std. Error** | **z value** | **LR Chisq** | **Df** | **Pr(>Chisq)** |
| Type | NA | NA | NA | 15.574 | 5 | **0.008** |
| Reads | 3.90E-06 | 2.53E-06 | 1.54 | 2.374 | 1 | 0.123 |
| Nr. of buildings | -0.048 | 0.019 | -2.465 | 6.085 | 1 | **0.013** |

**Table S10** Pairwise comparisons of habitat types for bacterial functional groups richness, obtained from estimated marginal means (EMMs) computed on the binomial GLM. Tukey-adjusted contrasts were calculated using the emmeans framework on the log-odds scale.

| **Potentially pathogenic bacteria** | | | | | | **Human-related bacteria** | | | | | | **Environmental bacteria** | | | | | |
| --- | --- | --- | --- | --- | --- | --- | --- | --- | --- | --- | --- | --- | --- | --- | --- | --- | --- |
| **Contrast** | **Estimate** | **SE** | **Df** | **z ratio** | **p value** | **Contrast** | **Estimate** | **SE** | **Df** | **z ratio** | **p value** | **Contrast** | **Estimate** | **SE** | **Df** | **z ratio** | **p value** |
| cave - pond | -1.201 | 0.188 | Inf | -6.385 | **<.0001** | cave - pond | -0.769 | 0.180 | Inf | -4.278 | **0.003** | cave - pond | -0.192 | 0.080 | Inf | -2.398 | 0.157 |
| cave - pool | 0.411 | 0.476 | Inf | 0.864 | 0.955 | cave - pool | 0.090 | 0.551 | Inf | 0.163 | 1.000 | cave - pool | -0.169 | 0.254 | Inf | -0.668 | 0.985 |
| cave - salt | -0.523 | 0.231 | Inf | -2.264 | 0.209 | cave - salt | -0.298 | 0.209 | Inf | -1.427 | 0.711 | cave - salt | -0.161 | 0.091 | Inf | -1.764 | 0.489 |
| cave - sea | -0.228 | 0.282 | Inf | -0.807 | 0.966 | cave - sea | 0.370 | 0.303 | Inf | 1.222 | 0.826 | cave - sea | 0.098 | 0.118 | Inf | 0.828 | 0.962 |
| cave - well | -0.903 | 0.249 | Inf | -3.626 | **0.004** | cave - well | -0.890 | 0.296 | Inf | -3.008 | **0.032** | cave - well | 0.173 | 0.151 | Inf | 1.143 | 0.863 |
| pond - pool | 1.612 | 0.477 | Inf | 3.378 | **0.010** | pond - pool | 0.859 | 0.506 | Inf | 1.696 | 0.535 | pond - pool | 0.023 | 0.233 | Inf | 0.098 | 1.000 |
| pond - salt | 0.678 | 0.170 | Inf | 3.977 | **0.001** | pond - salt | 0.472 | 0.157 | Inf | 2.998 | **0.033** | pond - salt | 0.031 | 0.070 | Inf | 0.448 | 0.998 |
| pond - sea | 0.973 | 0.312 | Inf | 3.121 | **0.022** | pond - sea | 1.139 | 0.345 | Inf | 3.297 | **0.013** | pond - sea | 0.290 | 0.144 | Inf | 2.012 | 0.335 |
| pond - well | 0.298 | 0.224 | Inf | 1.328 | 0.770 | pond - well | -0.121 | 0.241 | Inf | -0.502 | 0.996 | pond - well | 0.365 | 0.129 | Inf | 2.824 | 0.054 |
| pool - salt | -0.934 | 0.499 | Inf | -1.873 | 0.419 | pool - salt | -0.387 | 0.531 | Inf | -0.729 | 0.978 | pool - salt | 0.008 | 0.244 | Inf | 0.035 | 1.000 |
| pool - sea | -0.639 | 0.510 | Inf | -1.254 | 0.810 | pool - sea | 0.280 | 0.619 | Inf | 0.453 | 0.998 | pool - sea | 0.267 | 0.275 | Inf | 0.971 | 0.927 |
| pool - well | -1.315 | 0.493 | Inf | -2.664 | **0.083** | pool - well | -0.980 | 0.452 | Inf | -2.170 | 0.252 | pool - well | 0.342 | 0.203 | Inf | 1.689 | 0.539 |
| salt - sea | 0.295 | 0.343 | Inf | 0.861 | 0.956 | salt - sea | 0.667 | 0.363 | Inf | 1.838 | 0.441 | salt - sea | 0.259 | 0.152 | Inf | 1.704 | 0.529 |
| salt - well | -0.380 | 0.269 | Inf | -1.412 | 0.720 | salt - well | -0.593 | 0.282 | Inf | -2.100 | 0.287 | salt - well | 0.334 | 0.143 | Inf | 2.330 | 0.182 |
| sea - well | -0.676 | 0.318 | Inf | -2.123 | 0.275 | sea - well | -1.260 | 0.403 | Inf | -3.128 | 0.022 | sea - well | 0.075 | 0.185 | Inf | 0.405 | 0.999 |

**Table S12.** Analysis of deviance (Type II likelihood-ratio tests) for the Negative Binomial model evaluating total microbial richness within cave habitats. The model was fitted using richness as the response and light zone, sequencing depth (reads), and distance from the sea as predictors.

|  | **Estimate** | **Std. Error** | **z value** | **LR Chisq** | **Df** | **Pr(>Chisq)** |
| --- | --- | --- | --- | --- | --- | --- |
| Light | NA | NA | NA | 24.246 | 2 | **5.43E-06** |
| Reads | 2.77E-05 | 1.07E-05 | 2.589 | 6.451 | 1 | **0.011** |
| Distance from sea | -1.24E-03 | 4.45E-04 | -2.795 | 7.834 | 1 | **0.005** |

**Table S13** Pairwise Tukey-adjusted comparisons of light zones for microbial richness within caves, based on estimated marginal means (EMMs) from the Negative Binomial model. Contrasts were computed using the emmeans framework on the log scale.

| **Contrast** | **Estimate** | **SE** | **Df** | **z ratio** | **p value** |
| --- | --- | --- | --- | --- | --- |
| dark - light | 0,013 | 0,219 | Inf | 0,061 | 0,998 |
| dark - twilight | -0,950 | 0,235 | Inf | -4,046 | **0,0002** |
| light - twilight | -0,964 | 0,214 | Inf | -4,504 | **< 0.0001** |

**Table S14** PERMANOVA assessing the effects of light zone, sequencing depth (reads), and distance from the sea on total beta diversity and its components (nestedness and turnover) within cave habitats

| **Total** | **Df** | **SumOfSqs** | **R2** | **F** | **Pr(>F)** |
| --- | --- | --- | --- | --- | --- |
| Light | 2 | 0.930 | 0.243 | 1.114 | 0.200 |
| Reads | 1 | 0.345 | 0.090 | 0.826 | 0.837 |
| Distance from sea | 1 | 0.462 | 0.121 | 1.106 | 0.269 |
| Residual | 5 | 2.088 | 0.546 |  |  |
| Total | 9 | 3.825 | 1.000 |  |  |
| **Nestedness** | **Df** | **SumOfSqs** | **R2** | **F** | **Pr(>F)** |
| Light | 2 | 0.249 | 0.517 | 3.672 | 0.064 |
| Reads | 1 | 0.057 | 0.118 | 1.674 | 0.237 |
| Distance from sea | 1 | 0.006 | 0.013 | 0.179 | 0.776 |
| Residual | 5 | 0.170 | 0.352 |  |  |
| Total | 9 | 0.482 | 1.000 |  |  |
| **Turnover** | **Df** | **SumOfSqs** | **R2** | **F** | **Pr(>F)** |
| Light | 2 | 0.190 | 0.094 | 0.382 | 0.932 |
| Reads | 1 | 0.185 | 0.091 | 0.746 | 0.713 |
| Distance from sea | 1 | 0.412 | 0.203 | 1.661 | 0.165 |
| Residual | 5 | 1.240 | 0.612 |  |  |
| Total | 9 | 2.027 | 1.000 |  |  |

**Table S15** Analysis of deviance (Type II likelihood-ratio tests) for the model assessing phylogenetic diversity, fitted using a Gamma GLM with a log link.

|  | **Estimate** | **Std. Error** | **t value** | **LR Chisq** | **Df** | **Pr(>Chisq)** |
| --- | --- | --- | --- | --- | --- | --- |
| Type | NA | NA | NA | 14.7002 | 5 | **0.0117** |
| Reads | 2.39E-07 | 1.18E-05 | 0.0200 | 0.0004 | 1 | 0.9844 |
| Distance from sea | -4.51E-05 | 2.78E-04 | -0.1620 | 0.0274 | 1 | 0.8685 |
| Nr. of buildings | 0.1122 | 0.0870 | 1.2900 | 1.3207 | 1 | 0.2505 |

**Table S16** Pairwise comparisons of habitat types for phylogenetic diversity, obtained from estimated marginal means (EMMs) computed on the Gamma GLM. Tukey-adjusted contrasts were calculated using the emmeans framework on the log scale.

| **Contrast** | **Estimate** | **SE** | **Df** | **z ratio** | **p value** |
| --- | --- | --- | --- | --- | --- |
| sea - cave | -0.590 | 0.371 | 19 | -1.590 | 0.614 |
| sea - pool | 0.019 | 0.391 | 19 | 0.048 | 1.000 |
| sea - salt | 0.259 | 0.512 | 19 | 0.506 | 0.995 |
| sea - well | 0.783 | 0.424 | 19 | 1.847 | 0.461 |
| sea - pond | -0.005 | 0.857 | 19 | -0.005 | 1.000 |
| cave - pool | 0.608 | 0.290 | 19 | 2.098 | 0.329 |
| cave - salt | 0.849 | 0.635 | 19 | 1.337 | 0.762 |
| cave - well | 1.373 | 0.353 | 19 | 3.894 | **0.011** |
| cave - pond | 0.585 | 0.759 | 19 | 0.771 | 0.969 |
| pool - salt | 0.240 | 0.653 | 19 | 0.368 | 0.999 |
| pool - well | 0.764 | 0.379 | 19 | 2.016 | 0.370 |
| pool - pond | -0.023 | 0.776 | 19 | -0.030 | 1.000 |
| salt - well | 0.524 | 0.633 | 19 | 0.828 | 0.959 |
| salt - pond | -0.264 | 1.020 | 19 | -0.259 | 1.000 |
| well - pond | -0.788 | 0.697 | 19 | -1.130 | 0.863 |

**Table S17** PERMANOVA results partitioning phylogenetic β-diversity into total, turnover, and nestedness components.

| **Total** | **Df** | **SumOfSqs** | **R2** | **F** | **Pr(>F)** |
| --- | --- | --- | --- | --- | --- |
| Type | 5 | 2.130 | 0.245 | 1.432 | **0.012** |
| Distance from sea | 1 | 0.254 | 0.029 | 0.853 | 0.661 |
| Nr. of buildings | 1 | 0.181 | 0.021 | 0.607 | 0.922 |
| Reads | 1 | 0.414 | 0.048 | 1.390 | 0.115 |
| Richness | 1 | 0.374 | 0.043 | 1.258 | 0.194 |
| Residual | 18 | 5.356 | 0.615 |  |  |
| Total | 27 | 8.708 | 1.000 |  |  |
| **Nestedness** | **Df** | **SumOfSqs** | **R2** | **F** | **Pr(>F)** |
| Type | 5 | 1.070 | 0.301 | 1.938 | 0.067 |
| Distance from sea | 1 | 0.017 | 0.005 | 0.154 | 0.881 |
| Nr. of buildings | 1 | 0.048 | 0.014 | 0.437 | 0.694 |
| Reads | 1 | 0.107 | 0.030 | 0.968 | 0.391 |
| Richness | 1 | 0.323 | 0.091 | 2.925 | **0.049** |
| Residual | 18 | 1.987 | 0.559 |  |  |
| Total | 27 | 3.552 | 1.000 |  |  |
| **Turnover** | **Df** | **SumOfSqs** | **R2** | **F** | **Pr(>F)** |
| Type | 5 | 0.349 | 0.147 | 0.766 | 0.694 |
| Distance from sea | 1 | 0.142 | 0.060 | 1.558 | 0.332 |
| Nr. of buildings | 1 | 0.089 | 0.037 | 0.974 | 0.541 |
| Reads | 1 | 0.219 | 0.093 | 2.404 | 0.133 |
| Richness | 1 | -0.071 | -0.030 | -0.774 | 0.930 |
| Residual | 18 | 1.642 | 0.693 |  |  |
| Total | 27 | 2.370 | 1.000 |  |  |

**Figure S1 Composition of pathogenic, human-related, and environmental bacteria across habitat types.** Heatmaps show the mean relative abundance of the 10 most abundant genera within each subset across habitats. (**a**) Genera containing ASVs classified as potentially pathogenic bacteria. (**b**) Genera containing ASVs associated with human or wastewater sources. (**c**) Genera containing ASVs classified as environmental bacteria. Values represent mean relative abundance calculated relative to the total bacterial community of each sample

**Figure S2 Patterns of bacterial richness across environmental and anthropogenic gradients.** (**a**) Richness values across salinity categories: brackish seawater (bsw), fresh seawater (fsw), hypersaline seawater (hsw), and saline seawater (ssw); (**b**) Effect of light exposure on richness; (**c**) Richness across habitat types; (**d**) Relationship between richness and urbanization, shown as log-transformed number of buildings within 1 km

**Figure S3** **Partitioning of phylogenetic β-diversity within and across habitat types.** Density plots of pairwise β-diversity values for total β (top), turnover (middle), and nestedness (bottom) components. Distributions compare comparisons within the same habitat type (cyan) vs. across different habitats (orange)

**Figure S4** Dendrograms show similarity in composition across samples for: (**a**) the whole bacterial community, (**b**) pathobiome, (**c**) human-related and (**d**) environmental bacterial taxa

**Figure S5** Discriminability of habitats from one-vs-rest random forests across bacterial groups and taxonomic ranks. (**a**) ROC AUC. (**b**) Accuracy (1−OOB) for models trained separately for each habitat (columns: cave, pool, salt, sea, well) against the remaining habitats, using the entire community (“All”), Pathogens, Human-related, and Environmental subsets. Counts were aggregated to family, genus, and species; spring-fed pond was removed (n = 1). Each tile shows the metric value (10,000 trees; class-balanced sampling). Diverging red–white–blue scales emphasize performance relative to target thresholds (AUC midpoint = 0.85; Accuracy midpoint = 0.80): blue indicates better-than-threshold separability, red worse

**References**

1.	Martínez A, García-Gómez G, García-Herrero Á, et al (2019) Lanzarote and Chinijo Islands: An Anchialine UNESCO Global Geopark. In: Mateo E, Martínez-Frías J, Vegas J (eds) Lanzarote and Chinijo Islands Geopark: From Earth to Space. Springer International Publishing, Cham, pp 109–121

2.	Martinez A, Gonzalez BC, Núñez J, et al (2016) Guide to the anchialine ecosystems of Los Jameos del Agua and Túnel de la Atlántida. Cabildo de Lanzarote

3.	Trapero M, Santana Martel E (2025) Toponimia de Lanzarote. https://toplanzarote.ulpgc.es/. Accessed 17 Oct 2025

4.	Venables WN, Ripley BD (2002) Modern Applied Statistics with S, Fourth. Springer US

5.	Lüdecke D, Ben-Shachar MS, Patil I, et al (2021) performance: An R Package for Assessment, Comparison and Testing of Statistical Models. Journal of Open Source Software 6:3139. https://doi.org/10.21105/joss.03139

6.	Fox J, Weisberg S (2018) An R companion to applied regression. Sage publications

7.	Lenth RV (2025) emmeans: Estimated Marginal Means, aka Least-Squares Means. https://doi.org/10.32614/CRAN.package.emmeans

8.	Cardoso P, Rigal F, Carvalho JC (2015) BAT – Biodiversity Assessment Tools, an R package for the measurement and estimation of alpha and beta taxon, phylogenetic and functional diversity. Methods in Ecology and Evolution 6:232–236. https://doi.org/10.1111/2041-210X.12310

9.	Oksanen J, Simpson GL, Blanchet FG, et al (2025) vegan: Community Ecology Package

10.	R Core Team (2023) R: A Language and Environment for Statistical Computing

11.	Brooks M E, Kristensen K, Benthem K J ,van, et al (2017) glmmTMB Balances Speed and Flexibility Among Packages for Zero-inflated Generalized Linear Mixed Modeling. The R Journal 9:378. https://doi.org/10.32614/RJ-2017-066

12.	Katoh K, Rozewicki J, Yamada KD (2019) MAFFT online service: multiple sequence alignment, interactive sequence choice and visualization. Brief Bioinform 20:1160–1166. https://doi.org/10.1093/bib/bbx108

13.	Paradis E, Schliep K (2019) ape 5.0: an environment for modern phylogenetics and evolutionary analyses in R. Bioinformatics 35:526–528. https://doi.org/10.1093/bioinformatics/bty633

14.	Custodio E (2002) Aquifer overexploitation: what does it mean? Hydrogeology Journal 10:254–277. https://doi.org/10.1007/s10040-002-0188-6

15.	Izquierdo T (2014) Conceptual hydrogeological model and aquifer system classification of a small volcanic island (La Gomera; Canary Islands). CATENA 114:119–128. https://doi.org/10.1016/j.catena.2013.11.006

16.	Marín C, Luengo A (1994) El jardin de la sal. Ecotopía Ediciones Tenydea

17.	Masero JA (2003) Assessing alternative anthropogenic habitats for conserving waterbirds: salinas as buffer areas against the impact of natural habitat loss for shorebirds. Biodiversity and Conservation 12:1157–1173. https://doi.org/10.1023/A:1023021320448

18.	Saiz-Jimenez C (2012) Microbiological and environmental issues in show caves. World J Microbiol Biotechnol 28:2453–2464. https://doi.org/10.1007/s11274-012-1070-x

19.	Martínez A, Di Cesare A, Mari-Mena N, et al (2020) Tossed ‘good luck’ coins as vectors for anthropogenic pollution into aquatic environment. Environmental Pollution 259:113800. https://doi.org/10.1016/j.envpol.2019.113800

20.	Eshtawi T, Evers M, Tischbein B, Diekkrüger B (2016) Integrated hydrologic modeling as a key for sustainable urban water resources planning. Water Research 101:411–428. https://doi.org/10.1016/j.watres.2016.05.061

21.	Moore WS (2010) The effect of submarine groundwater discharge on the ocean. Ann Rev Mar Sci 2:59–88. https://doi.org/10.1146/annurev-marine-120308-081019

22.	Santos IR, Chen X, Lecher AL, et al (2021) Submarine groundwater discharge impacts on coastal nutrient biogeochemistry. Nat Rev Earth Environ 2:307–323. https://doi.org/10.1038/s43017-021-00152-0

23.	Wilkens H, Iliffe TM, Oromí P, et al (2009) The Corona lava tube, Lanzarote: geology, habitat diversity and biogeography. Mar Biodiv 39:155–167. https://doi.org/10.1007/s12526-009-0019-2
